# ErbB3 is required for hyperaminoacidemia-induced pancreatic α cell hyperplasia

**DOI:** 10.1016/j.jbc.2024.107499

**Published:** 2024-06-27

**Authors:** Qi Kang, Jianxin Jia, E Danielle Dean, Hang Yuan, Chunhua Dai, Zhehui Li, Fuquan Jiang, Xiao-Kun Zhang, Alvin C. Powers, Wenbiao Chen, Mingyu Li

**Affiliations:** 1 School of Pharmaceutical Sciences and School of Life Sciences, Xiamen University, Xiamen, China; 2 Fujian Provincial Key Laboratory of Innovative Drug Target Research, School of Pharmaceutical Sciences, Xiamen University, Xiamen, China; 3 Departments of Molecular Physiology and Biophysics, Vanderbilt University School of Medicine, Nashville, Tennessee, USA; 4 Division of Diabetes, Endocrinology, and Metabolism, Department of Medicine, Vanderbilt University Medical Center, Nashville, Tennessee, USA; 5 VA Tennessee Valley Healthcare System, Nashville, Tennessee, USA; 6 State Key Laboratory of Vaccines for Infectious Diseases, Xiang An Biomedicine Laboratory, Xiamen University, Xiamen, China

**Keywords:** hyperaminoacidemia, glucagon receptor, α cell, α cell hyperplasia, diabetes

## Abstract

Blood amino acid levels are maintained in a narrow physiological range. The pancreatic α cells have emerged as the primary aminoacidemia regulator through glucagon secretion to promote hepatic amino acid catabolism. Interruption of glucagon signaling disrupts the liver–α cells axis leading to hyperaminoacidemia, which triggers a compensatory rise in glucagon secretion and α cell hyperplasia. The mechanisms of hyperaminoacidemia-induced α cell hyperplasia remain incompletely understood. Using a mouse α cell line and *in vivo* studies in zebrafish and mice, we found that hyperaminoacidemia-induced α cell hyperplasia requires ErbB3 signaling. In addition to mechanistic target of rapamycin complex 1, another ErbB3 downstream effector signal transducer and activator of transcription 3 also plays a role in α cell hyperplasia. Mechanistically, ErbB3 may partner with ErbB2 to stimulate cyclin D2 and suppress p27 *via* mechanistic target of rapamycin complex 1 and signal transducer and activator of transcription 3. Our study identifies ErbB3 as a new regulator for hyperaminoacidemia-induced α cell proliferation and a critical component of the liver–α cells axis that regulates aminoacidemia.

Amino acids are building blocks of proteins. Sufficient blood amino acid supply is essential for cell growth and proliferation. Indeed, blood amino acid levels (aminoacidemia) are maintained in a narrow physiological range, and deviation from the range is usually a sign of physiological abnormality. Although our understanding of cellular amino acid sensing and adaptation has tremendously improved in recent years, our knowledge of aminoacidemia control is relatively poor.

Glucagon has emerged as the primary negative regulator of aminoacidemia. Glucagon was discovered 100 years ago as a positive regulator of glycemia from the pancreas (1). Secreted from pancreatic α cells, glucagon is vital for the regulation of glucose homeostasis, lipid metabolism, and amino acid metabolism (2, 3). It is best known as the counterregulatory hormone for insulin in glycemic control. Glucagon stimulates hepatic glucose output by glycogenolysis and gluconeogenesis in the liver through glucagon receptor (GCGR) (4). Increased glucagon function is thought to be essential for the development of type 1 and type 2 diabetes (5, 6). GCGR antagonism is thus proposed as a diabetes treatment (5, 6). Accordingly, genetic suppression of glucagon signaling in mice prevents diabetes and GCGR antagonism improves glycemic control in both diabetic rodents and humans (7, 8, 9, 10, 11, 12). However, acute and chronic GCGR signaling disruption causes α cell hyperplasia, among other undesired effects (13, 14, 15). Chronic α cell hyperplasia in germline GCGR-deficient individuals eventually leads to glucagonoma in mice and humans (16). The α cell hyperplasia results from hyperaminoacidemia consequent to reduced hepatic amino acid catabolism (17, 18, 19), revealing a liver–α cell axis (20, 21, 22, 23). Conversely, intravenous glucagon or GCGR agonism causes hypoaminoacidemia (24, 25, 26). Therefore, glucagon is a negative controller of aminoacidemia.

How hyperaminoacidemia induces α cell proliferation remains incompletely understood. Hyperaminoacidemia, particularly glutamine, alanine, and arginine, stimulates α cell proliferation in an mechanistic target of rapamycin complex 1 (mTORC1)-dependent manner (18, 19, 27). In mice, mTORC1 activation also induces the small neutral amino acid transporter *Slc38a5* in α cells, further enhancing pancreatic α cell hyperplasia (18, 19). In addition to mTORC1 activation by intracellular amino acids, increased extracellular amino acids also activate the Ca^2+^-sensing receptor (CaSR)–G protein subtypes Gq (Gq)–extracellular signal-regulated kinase1/2 (ERK1/2) pathway (28). Activation of the two pathways causes α cell proliferation in the absence of hyperaminoacidemia (28). Whether hyperaminoacidemia-induced α cell proliferation requires other signaling pathway(s) is unknown.

The erythroblastic leukemia viral oncogene homolog (ErbB) receptor family, belongs to the subclass Ⅰ receptor tyrosine kinase superfamily, which contains four members (ErbB1 to ErbB4) in mammals. The ErbB family is expressed in many tissues and plays a pivotal role in the growth and development of numerous organs such as the mammary gland, heart, and central nervous system. In the pancreatic islet cells, several studies have demonstrated a role for ErbB signaling in the development and lineage determination of endocrine cells (29, 30). The dysregulated expression or overactivation of ErbB is correlated with tumorigenesis of many human organs. In general, the ErbB signal transduction is initiated by ligand-induced receptor dimerization, which results in activation of the intrinsic kinase domain and cross-phosphorylation of the dimer partners, creating the docking sites for downstream signaling molecules (31). Despite its well-known role in cell proliferation, the role of ErbB signaling in hyperaminoacidemia-induced α cell proliferation has never been reported and remains undetermined.

In this study, we took an unbiased approach to identify other required component(s) for hyperaminoacidemia-induced α cell proliferation. We established a GCGR KO model in zebrafish, which displays hyperaminoacidemia, α cell hyperplasia, and mild hypoglycemia as in mammals (28, 32, 33, 34). Taking advantage of the chemical tractability of zebrafish, we performed a small molecule screen and identified the ErbB family receptor tyrosine kinases prevented α cell hyperplasia in *gcgr*-deficient fish. Further genetic analyses in αTC1-6 cell line, zebrafish, and mice revealed that hyperaminoacidemia activates ErbB3. Mechanistically, activation of ErbB3 upregulates cyclin D2 while downregulates p27, promoting cell cycle progression.

## Results

### Small molecule screening in zebrafish implicates ErbB signaling in *gcgr* deficiency–induced α cell hyperplasia

**Figure 1:**
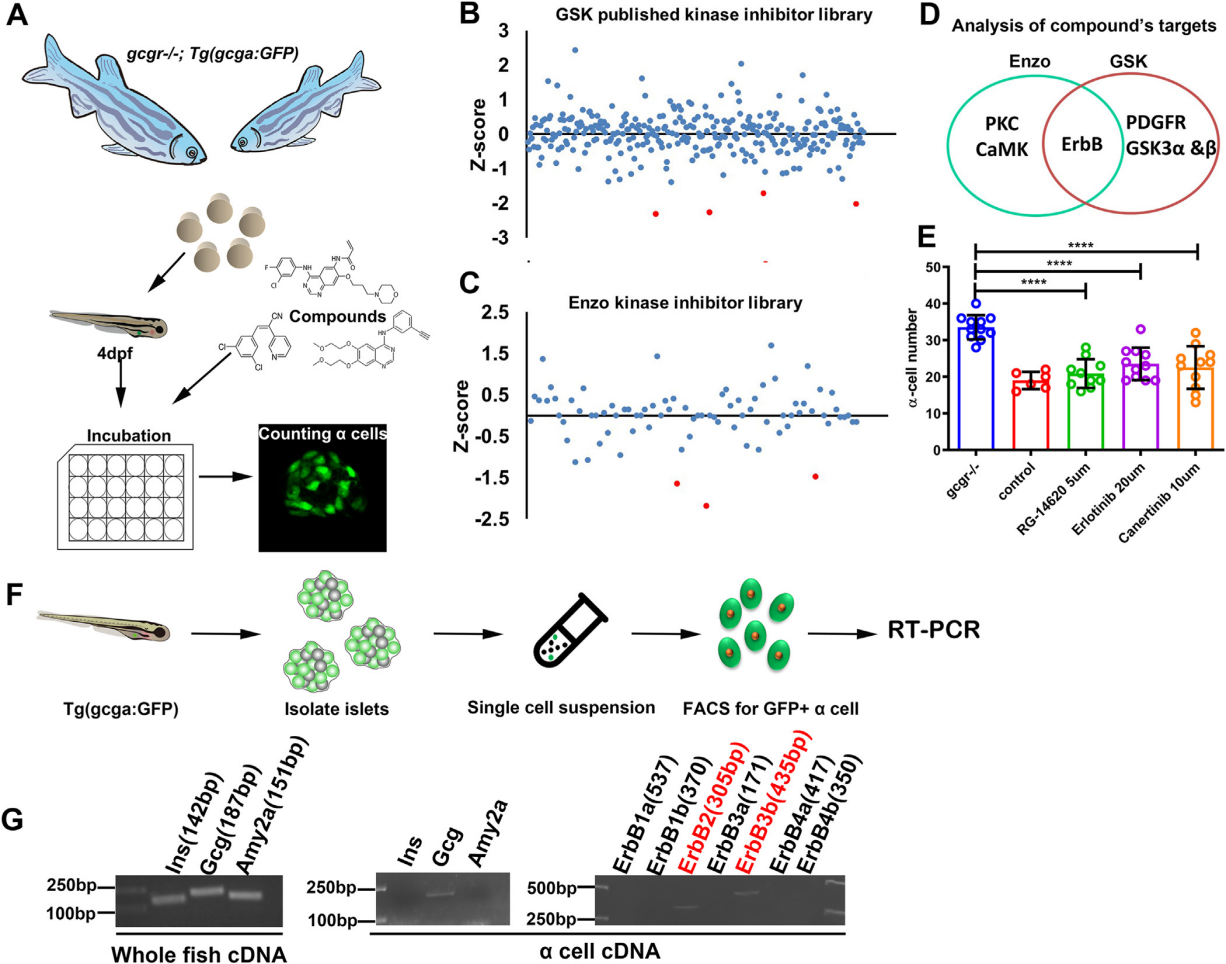
**Identification the role of ErbB2 and ErbB3 in *gcgr* deficiency zebrafish.***A,* schematic of small molecule inhibitor screening using zebrafish reporter line *gcgr−/−; Tg (gcga: GFP)*. *B* and *C,* Z-score analysis of the effect on α cell number in 7 dpf larvae by individual compounds from the GSK kinase inhibitor library (*B*) or Enzo kinase inhibitor library (*C*), and the *red dots* indicate hit compounds. *D, Venn diagram* of the target kinases of the hit compounds from (*B* and *C*) indicating ErbB as a strong candidate. *E,* effect of several other ErbB inhibitors on α cell number in *gcgr*^*−/−*^ larvae (n = 6–11), and the cell number was analyzed using one-way ANOVA with a Bonferroni post hoc test. Data are presented as mean ± SD. ∗∗∗∗*p* < 0.0001. *F,* schematic of zebrafish α cells isolation from transgenic line *Tg(gcga: GFP)* by FACS. *G,* gene expression profile of all *erbB* members in the zebrafish α cells. Comparison of PCR results from whole fish and α cells is included as a quality control of α cell purity. dpf, days post fertilization; FACS, fluorescence-activated cell sorting; GCGR, glucagon receptor.

To identify the driving factor of the α cell hyperplasia, we performed drug screening using the *gcgr*^*−/−*^ zebrafish model. We screened two kinase inhibitor libraries, one developed by GSK (349 compounds) and the other, by Enzo (80 compounds) for inhibitors of α cell hyperplasia. Four days post fertilization (dpf) *gcgr*^*−/−*^*; Tg(gcga: GFP)* zebrafish larvae were incubated with individual compounds for 3 days. The number of GFP-labeled α cells in each larva was counted (Fig. 1*A*). Four compounds (GSK1030061A, GW869810X, GW439255X, and SB-678557-A) from the GSK library and three compounds (GF 109203X, KN-62, and RG-1462) from the Enzo library significantly decreased α cell number in *gcgr*^*−/−*^*; Tg(gcga:GFP)* zebrafish larvae (Fig. 1, *B* and *C* and Table. S1). After analyzing the targets of these seven compounds, we found that ErbB family was the common target from the two screens (Fig. 1*D*). To further confirm our screen results, we treated *gcgr*^*−/−*^*; Tg(gcga: GFP)* with three other ErbB inhibitors, RG-14620, erlotinib, and canertinib. All these three ErbB inhibitors significantly decreased α cell number (Fig. 1*E*). These data suggested that ErbB signaling plays an important role in α cell hyperplasia in *gcgr*^*−/−*^ zebrafish.

ErbB family consists of multiple members in zebrafish. To determine which family members are expressed in α cells, we sorted the α cells from *Tg(gcga: GFP)* larvae (Fig. 1*F*) and performed the RT-PCR to survey the expression of all seven members (*erbB1a*, *erbB1b*, *erbB2*, *erbB3a*, *erbB3b*, *erbB4a*, and *erbB4b*). As shown in Figure 1*G*, only *erbB2* and *erbB3b* were detectable. These results indicated that ErbB signaling is necessary for α cell hyperplasia in *gcgr*-deficient zebrafish, and ErbB2 and ErbB3 are the candidate mediators in zebrafish.

### Inhibition of ErbB2 and ErbB3 diminishes α cell hyperplasia in *gcgr*-deficient zebrafish

**Figure 2:**
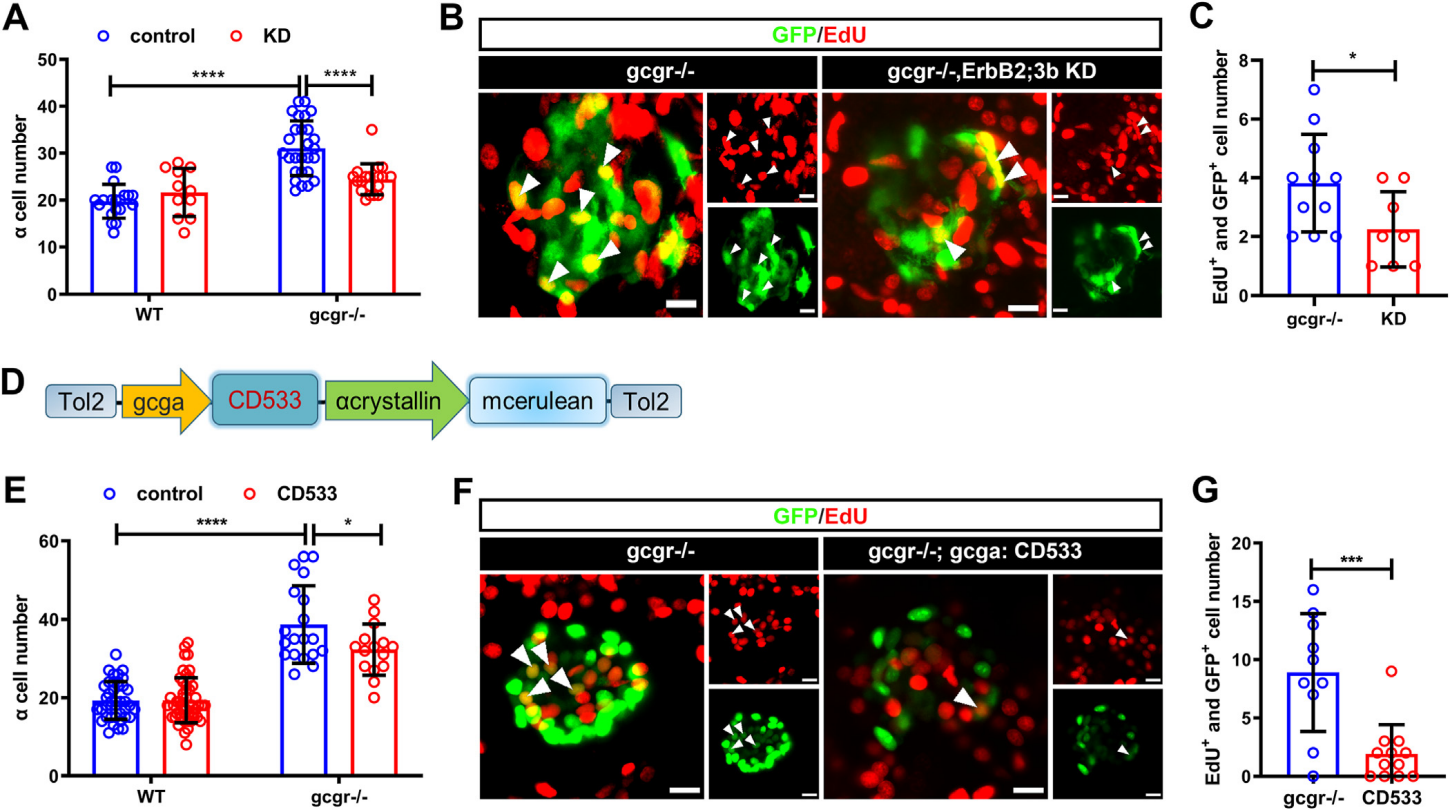
**Genetic validation of the role for ErbB2 and ErbB3 in *gcgr*-deficiency zebrafish.***A–C,* effect of *erbB2* and *erbB3b* knockdown on α cell proliferation. *A,* quantification of α cell number at 7 dpf in control and *gcgr*^*−/−*^ larvae with or without *erbB2* and *erbB3b* knockdown. All fish carry *Tg(gcga: GFP)* for α cell quantification (n = 11–27), and the cell number was analyzed using two-way ANOVA with a Bonferroni post hoc test. Data are presented as mean ± SD. ∗ *p* < 0.05 and ∗∗∗∗*p* < 0.0001. *B* and *C,* representative confocal projections (*B*) and quantification (*C*) of EdU labeling at 7 dpf in control and *gcgr*^*−/−*^ larvae with or without *erbB2* and *erbB3b* knockdown. All fish carry *Tg(gcga: GFP)* for α cell quantification (n = 8–11) and the cell number was analyzed using unpaired two-tailed *t* test. Data are presented as mean ± SD, ∗ *p* < 0.05. The scale bar represents 10 μm. *D,* schematic of the *Tg(gcga: CD533, LC)* transgene used to express a dominant-negative ERBB1 in α cells under the control of zebrafish glucagon promoter. The linked α crystallin promoter: mCerulean is used for genotyping. *E*–*G*, effect of *CD533* on the α cell proliferation. *E,* quantification of the α cell number at 7 dpf in control and *gcgr*^*−/−*^ larvae with or without *Tg(gcga: CD533)* (n = 15–48), the cell number was analyzed using two-way ANOVA with a Bonferroni post hoc test. Data are presented as mean ± SD. ∗ *p* < 0.05 and ∗∗∗∗*p* < 0.0001. *F* and *G,* representative confocal projections (*F*) and quantification (*G*) of EdU labeling at 7 dpf in control and *gcgr*^*−/−*^ larvae with or without *Tg(gcga: CD533, LC)* (n = 10–12), and the cell number was analyzed using unpaired two-tailed *t* test. Data are presented as mean ± SD, ∗∗∗ *p* < 0.001. *Arrows* indicate EdU and GFP double positive cells. The scale bar represents 10 μm. dpf, days post fertilization; EdU, 5-ethynyl-2-deoxyuridine; GCGR, glucagon receptor.

To confirm the role of ErbB2 and ErbB3b in zebrafish, we generated *erbB2/3b* crispants by coinjecting Cas9 protein with two sgRNAs each for *erbB2* and *erbB3b* into WT or *gcgr*-deficient zebrafish zygotes. As shown in Figure 2, *A*–*C*, *erbB2/3b* knockdown did not affect the α cell numbers in the WT group but reduced both α cell numbers and α cell proliferation in *gcgr*^*−/−*^ larvae, consistent with an essential for ErbB2/3b in α cell hyperplasia. To determine whether ErbB2/3b acts cell autonomously, we generated a transgenic zebrafish line with α cell–specific overexpression of a dominant negative ErbB1 (CD533) (Fig. 2*D*). CD533 lacks 533 residuals in the C terminus of ErbB1. It can block signaling from all ErbB receptor tyrosine kinases (35, 36, 37, 38). *gcgr*^*−/−*^*; Tg(gcga:CD533)* zebrafish larvae had significantly decreased α cell number (Fig. 2*E*). This decrease is at least partially due to lower proliferation as fewer α cells were labeled by EdU (Fig. 2, *F* and *G*). These results suggest a cell-autonomous role for ErbB2/ErbB3b in α cell hyperplasia in *gcgr*^*−/−*^ zebrafish.

### *Gcgr*^−/−^ mice increased ErbB3 activity in α cells

**Figure 3:**
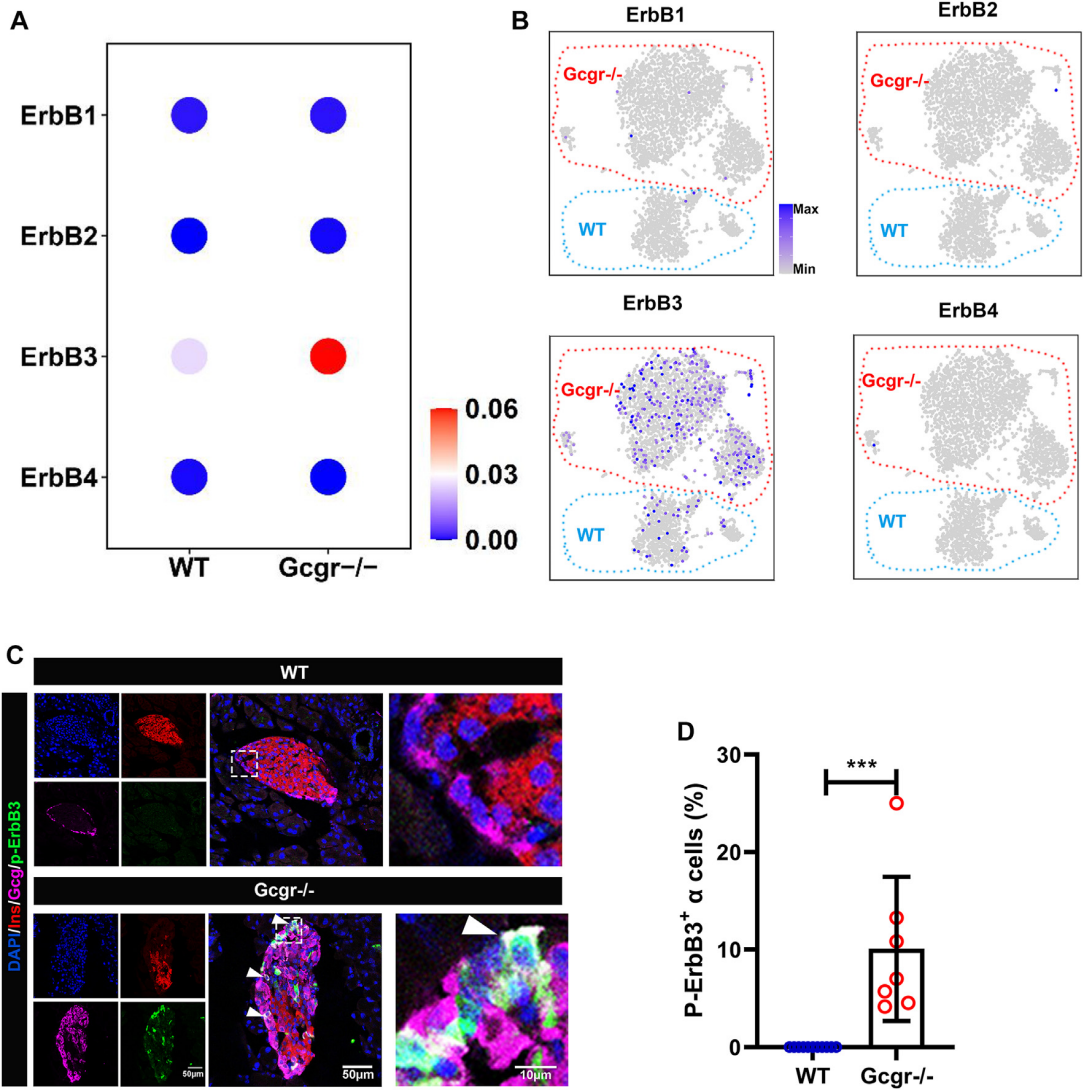
**ErbB3 activated in *Gcgr***^***−/−***^**mouse α cells.***A* and *B,* the *ErbBs* expression levels (*A*) and distribution (*B*) in WT *versus Gcgr*^*−/−*^ mouse α cells shown by heatmap and t-distributed stochastic neighbor embedding plots. Data was analyzed based on the single-cell sequencing data from our lab (GSE253271). Scale ranges correspond to the gene expression as indicated. *C* and *D,* representative images of p-ErbB3 (*C*) and quantification (*D*) of immunofluorescence in pancreatic islets from WT and *Gcgr*^*−/−*^ mice. *Arrowheads* point to representative p-ErbB3–positive α cells. n = 7 to 11. The p-ErbB3–positive α cells ratio was analyzed using unpaired two-tailed *t* test. Data are presented as mean ± SD. ∗∗∗*p* < 0.001. GCGR, glucagon receptor.

Hyperplasia is a conserved α cell response to interruption of glucagon signaling in zebrafish, mice, and humans. To investigate whether ErbBs are expressed in mammalian α cells, we first analyzed the single-cell RNA-seq data from WT and *Gcgr*^*−/−*^ mice islet α cells generated by our group (39). The results showed that ErbB3 is the dominant member of ErbB family in mouse α cells. ErbB1, ErbB2, and ErbB4 were undetectable (Fig. 3, *A* and *B*). Moreover, the ErbB3 expression level and ErbB3-positive cell ratio (11.0% *versus* 3.5%) were increased in islet α cells from *Gcgr*^*−/−*^ mice (Fig. 3*B*). The immunofluorescence staining results showed that ErbB3, but not ErbB1, ErbB2 or ErbB4, was indeed expressed in a subset of α cells in both WT and *Gcgr*^*−/−*^ mice islets (Fig. S1). Remarkably, our results also revealed that p-ErbB3 (the active form of ErbB3, phosphorylated ErbB3) stain was strong in a subset of α cells in *Gcgr*^*−/−*^ mice while absent in WT islets. (Fig. 3, *C* and *D*). These indicated that ErbB3 may be the key factor for α cell proliferation in *Gcgr*^*−/−*^ mice.

**Figure 4:**
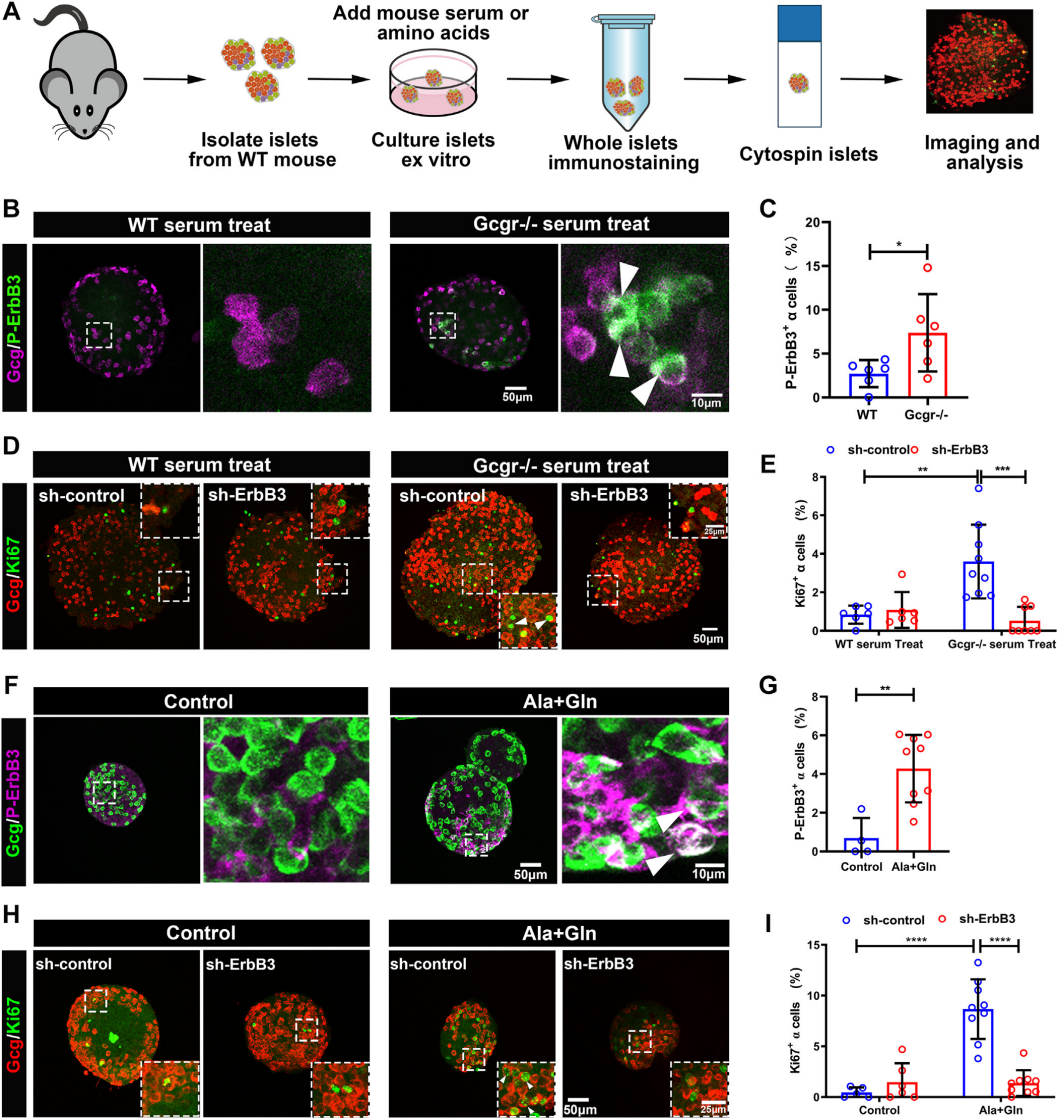
**ErbB3 is essential for *Gcgr***^***−/−***^**mouse serum or high amino acid stimulated α cell proliferation.***A,* schematic for *ex vivo* islet culture with mouse serum or high amino acids stimulation and α cell proliferation quantification assay. *B* and *C,* representative immunofluorescence images (*B*) and quantification (*C*) of islets cultured medium with WT and *Gcgr*^*−/−*^ serum. *Arrowheads* point to representative p-ErbB3–positive α cells (n = 6), and p-ErbB3–positive α cells ratio was analyzed using the unpaired two-tailed *t* test. Data are presented as mean ± SD, ∗*p* < 0.05. *D* and *E*, representative immunofluorescence images (*D*) and quantification (*E*) of islets transduced with sh-ErbB3 or control adenovirus and cultured in medium with WT or *Gcgr*^*−/−*^ serum (n = 6–9). *Arrowheads* point to representative proliferating α cells (Ki67 and glucagon double positive), and the Ki67-positive α cells ratio was analyzed using two-way ANOVA with a Bonferroni post hoc test. Data are presented as mean ± SD. ∗∗ *p* < 0.01 and ∗∗∗*p* < 0.001. *F* and *G,* representative immunofluorescence images (*F*) and quantification (*G*) of islets cultured in the control medium or medium supplemented with glutamine and alanine. *White arrowheads* indicate representative p-ErbB3–positive α cells (n = 4–9), the p-ErbB3–positive α cells ratio was analyzed using the unpaired two-tailed *t* test. Data are presented as mean ± SD, ∗∗*p* < 0.01. *H* and *I,* representative immunofluorescence images (*H*) and quantification (*I*) of islets transduced by sh-ErbB3 or control adenovirus and cultured in control medium or medium supplemented with glutamine and alanine, and *white arrowheads* indicate representative Ki67-positive α cells (n = 5–9). The Ki67-positive α cells ratio was analyzed using two-way ANOVA with a Bonferroni post hoc test. Data are presented as mean ± SD. ∗∗∗∗*p* < 0.0001. GCGR, glucagon receptor.

To further explore the role of ErbB3 in the α cell hyperplasia, we employed the *ex vivo* primary islet culture. We cultured WT islets with serum from *Gcgr*^−/−^ or WT mice to observe whether ErbB3 was activated in α cells as *in vivo*. As shown in Figure 4, *B* and *C*, *Gcgr*^*−/−*^ serum significantly increased the percentage of p-ErbB3-positive α cells compared to the control serum. These data indicated that GCGR deficiency induces ErbB3 expression and activation in α cells.

### *ErbB3* knockdown suppresses *gcgr*^*−/−*^ serum or high amino acids induced α cell proliferation *ex vivo*

Since GCGR deficiency activates ErbB3 in α cells, we determined whether ErbB3 is essential for mouse α cell proliferation. To assess the role of ErbB3, we again used the primary mouse islets first. We employed adenovirus-mediated shRNA expression to knockdown *ErbB3* in cultured islets (Fig. S2, *A* and *B*). Immunofluorescence results indicated efficient depletion of ErbB3 by virus expressing sh-ErbB3 but not by virus with control shRNA (Fig. S2*B*). Combining with the *ex vivo* islet culture assay (Fig. 4*A*), we cultured transduced islets with serum from *Gcgr*^−/−^ or WT mice. As shown in Figure 4, *D* and *E*, *ErbB3* knockdown by sh-ErbB3 significantly diminished *Gcgr*^*−/−*^ serum-induced α cell proliferation. Moreover, *ErbB3* knockdown did not affect α cell proliferation in WT serum-treated islets (Fig. 4, *D* and *E*).

To further explore the role of ErbB3 in the hyperaminoacidemia-induced α cell hyperplasia, we supplied a high level of amino acids (4 mM glutamine and 4 mM alanine) in the primary mouse islets culture. As shown in Figure 4, *F* and *G*, high amino acids medium also increased p-ErbB3-positive α cells in the cultured islets. To determine whether ErbB3 is necessary for the high amino acids induced α cell proliferation, we cultured islets in the high amino acid medium with control shRNA or ErbB3-shRNA. High amino acids increased Ki67-positive α cells in the control shRNA group (Fig. 4, *H* and *I*). However, islets expressing sh-ErbB3 had significantly decreased Ki67-positive α cells compared to control (Fig. 4, *H* and *I*). These data indicated that ErbB3 is required for α cell proliferation in *Gcgr*^*−/−*^ serum or high amino acid medium.

### ErbB3 is essential for high amino acid stimulated αTC1-6 cell proliferation and is involved in mTOR activation

**Figure 5:**
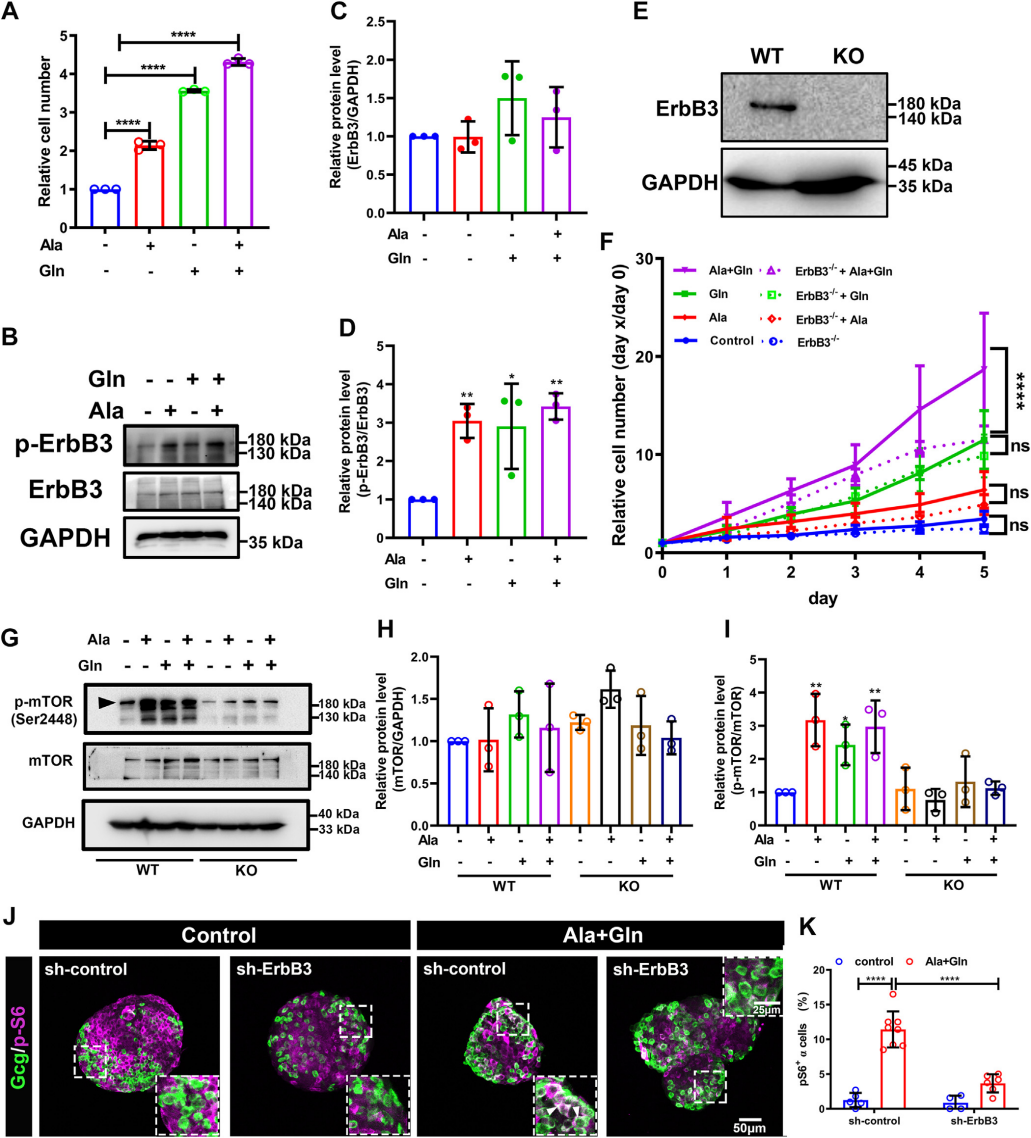
**ErbB3 is essential for high amino acid stimulated αTC1-6 proliferation and involved in mTOR activation.***A,* relative αTC1-6 cell number after 5 days in control medium and medium supplemented with alanine, glutamine, or both (n = 3), the relative cell number was analyzed using one-way ANOVA with a Bonferroni post hoc test. Data are presented as mean ± SD. ∗∗∗∗*p* < 0.0001. *B*–*D,* the representative image (*B*) and quantification (*C* and *D*) of relative ErbB3/GAPDH and p-ErbB3/ErbB3 levels in αTC1-6 after high amino acids treatment, and the relative protein level was analyzed using one-way ANOVA with a Bonferroni post hoc test. Data are presented as mean ± SD. ∗*p* < 0.05 and ∗∗*p* < 0.01. *E,* Western blot analysis of ErbB3 in WT and *ErbB3 KO* αTC1-6 cells. *F,* growth curve of WT (*solid lines*) and *ErbB3* KO (*dashed lines*) αTC1-6 cells in control or high amino acids medium, and the relative cell number was analyzed using two-way ANOVA with a Bonferroni post hoc test. Data are presented as mean ± SD. ∗∗∗∗*p* < 0.0001. *G–I,* the representative images (*G*) and quantification (*H* and *I*) of total mTOR/GAPDH and p-mTOR/mTOR levels in WT and *ErbB3* KO αTC1-6 cells cultured in control or high amino acids medium. The *black arrowhead* indicates the relevant bands, and the relative protein level was analyzed using one-way ANOVA with a Bonferroni post hoc test. Data are presented as mean ± SD. ∗*p* < 0.05 and ∗∗*p* < 0.01. *J* and *K,* representative immunofluorescence images (*J*) and quantification (*K*) of islets transduced with sh-ErbB3 or control adenovirus and cultured in the control medium or medium supplemented with glutamine and alanine. *White arrowheads* indicate representative p-S6–positive α cells (n = 4–8), and the p-S6–positive α cell ratio was analyzed using two-way ANOVA with a Bonferroni post hoc test. Data are presented as mean ± SD. ∗∗∗∗*p* < 0.0001.

To determine the molecular mechanism of ErbB3 mediates hyperaminoacidemia induced α cell proliferation, we used αTC1-6, a commonly used mouse α cell line. Like islet α cells, *Gcgr*^*−/−*^ serum also increased αTC1-6 cell proliferation (Fig. S3). These results suggested that αTC1-6 cell line is a suitable model for understanding amino acid–induced α cell proliferation. Moreover, medium with high glutamine or alanine significantly increased αTC1-6 cell growth compared to control medium (Fig. 5*A*). In addition, a combination of high glutamine and high alanine had an additive effect on αTC1-6 cell growth (Fig. 5*A*). Accordingly, high glutamine or alanine medium increased the p-ErbB3 level but not the ErbB3 protein level (Fig. 5, *B*–*D*). To confirm that ErbB3 is necessary for the high amino acid–induced growth, we generated an *ErbB3* KO subclone of αTC1-6 cells using CRISPR/Cas9 that has undetectable expression by Western blotting analysis (Figs. 5*E* and S4). The growth of *ErbB3* KO cells was not different from WT cells in control medium (Fig. 5*F*). However, loss of ErbB3 significantly suppressed high amino acid–induced αTC1-6 cell growth (Fig. 5*F*). These data suggested that ErbB3 is essential for glutamine and alanine induced α cell proliferation in αTC1-6 cells.

Although it is commonly assumed that hyperaminoacidemia drives mTORC1 activation primarily through increasing intracellular amino acids, we tested whether ErbB3 is important for mTORC1 activation under hyperaminoacidemia. As shown in Figure 5, *G*–*I*, high glutamine and alanine, either alone or in combination, significantly increased p-mTOR levels in WT αTC1-6 cells as expected, but not in the *ErbB3* knockout cells. Similarly, the high amino acid medium increased the percentage of pS6-positive α cells in islets transduced by the control shRNA as expected, indicative of mTORC1 activation (Fig. 5, *J* and *K*). However, the increase was significantly reduced in islets-expressing sh-ErbB3 (Fig. 5, *J* and *K*). Therefore, ErbB3 is necessary for full mTORC1 activation by the high amino acid medium *in vitro*.

### STAT3 plays a role in high amino acid–induced α cell proliferation

**Figure 6:**
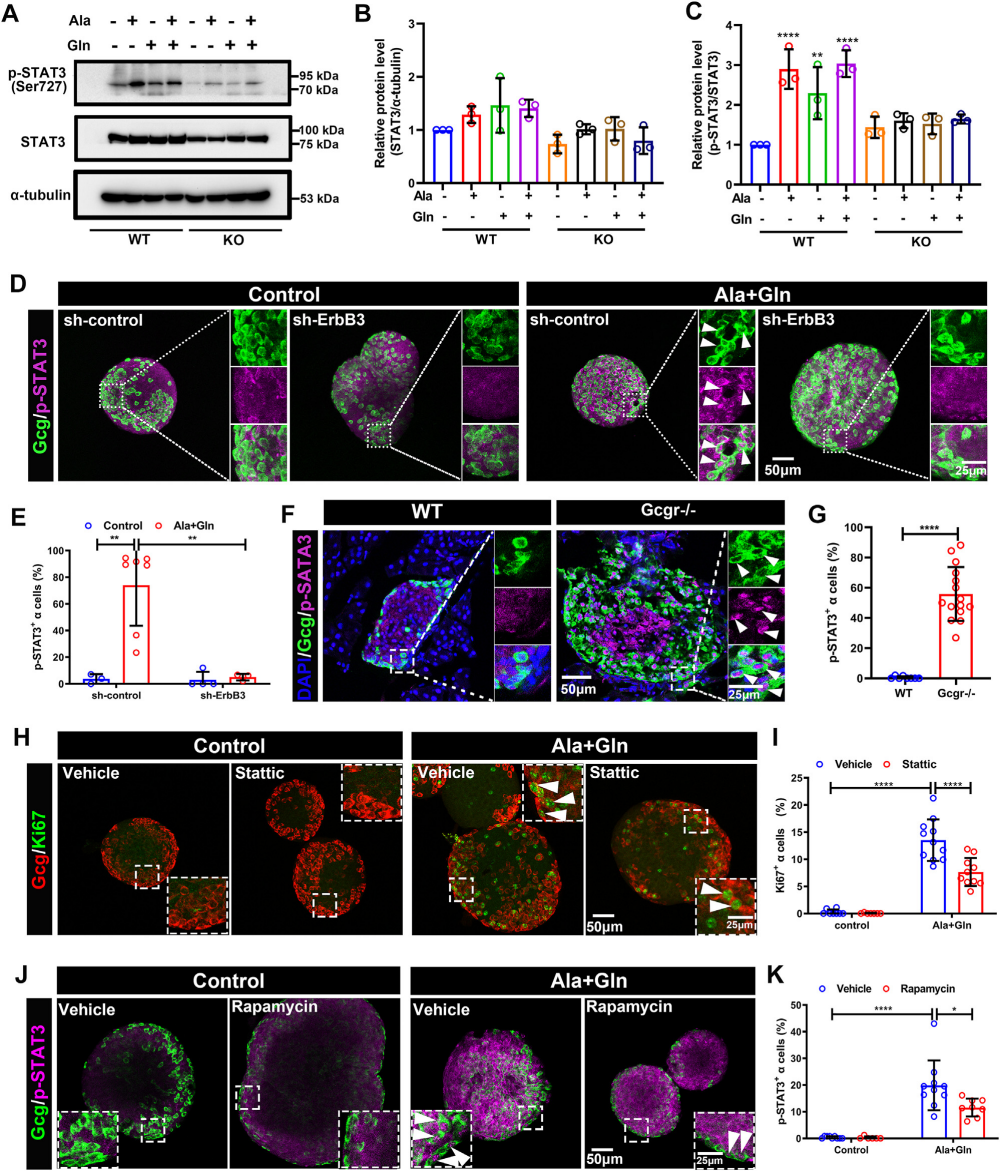
**STAT3 plays an important role in high amino acid induced α cell proliferation.***A*–*C,* representative images (*A*) and quantification (*B* and *C*) of total STAT3/α-tubulin and p-STAT3/STAT3 levels in WT and *ErbB3* KO αTC1-6 cells cultured in control or high amino acids medium, the relative protein level was analyzed using one-way ANOVA with a Bonferroni post hoc test. Data are presented as mean ± SD. ∗∗*p* < 0.01 and ∗∗∗∗*p* < 0.0001. *D* and *E,* representative immunofluorescence images (*D*) and quantification (*E*) of islets transduced by sh-ErbB3 or control adenovirus and cultured in control or glutamine plus alanine contained medium, *white arrowheads* indicate representative p-STAT3–positive α cells (n = 3–7), and the p-STAT3–positive α cells ratio was analyzed using two-way ANOVA with a Bonferroni post hoc test. Data are presented as mean ± SD. ∗∗*p* < 0.01. *F* and *G,* representative immunofluorescence images of p-STAT3 (*F*) and quantification (*G*) of the percentage of p-STAT3–positive α cells in pancreatic islets of WT and *Gcgr*^*−/−*^ mice (n = 8–15), and the p-STAT3–positive α cell ratio was analyzed using unpaired two-tailed *t* test. Data are presented as mean ± SD. ∗∗∗∗*p* < 0.0001. *H* and *I,* representative immunofluorescence images (*H*) and quantification (*I*) of islets cultured in the control medium or medium supplemented with glutamine and alanine in the presence of 1 μM Stattic or vehicle (n = 7–11), the Ki67-positive α cells ratio was analyzed using two-way ANOVA with a Bonferroni post hoc test. Data are presented as mean ± SD. ∗∗∗∗*p* < 0.0001. *White arrowheads* indicate representative Ki67-positive α cells. *J* and *K,* representative single optical section of islet immunofluorescence images (*J*) and quantification (*K*) of islets cultured in control medium or medium supplemented with glutamine and alanine in the presence of 30 nM rapamycin or vehicle (n = 6–10). *White arrowheads* indicate representative p-STAT3–positive α cells. The p-STAT3–positive α cells ratio was analyzed using two-way ANOVA with a Bonferroni post hoc test. Data are presented as mean ± SD. ∗ *p* < 0.05, ∗∗∗∗*p* < 0.0001. GCGR, glucagon receptor; STAT3, signal transducer and activator of transcription 3.

ErbB signaling stimulates multiple pathways including Ras/ERK1/2, Akt/mTOR, and Jak/signal transducer and activator of transcription 3 (Stat3) (40, 41, 42). The role of ERK1/2 and mTORC1, but not Stat3, has been reported (17, 18, 19, 28). ErbB3 or ErbB2/ErbB3 can activate Stat3 directly. The ErbB3–Stat3 pathway regulates many processes such as tumorigenesis (41, 43, 44), epithelial-mesenchymal transition (45), and drug resistance (46), to name a few. ErbB3 can also activate Stat3 *via* ERK1/2 (47) and mTORC1 (48, 49, 50). Hence, we assessed STAT3 activation and its role in high amino acid–induced α cell proliferation. As shown in Figure 6, *A*–*C*, high glutamine and alanine, either alone or in combination, increased p-STAT3 levels in WT αTC1-6 cells (Fig. 6, *A*–*C*). This effect was abolished in the *ErbB3* KO cells (Fig. 6, *A*–*C*). Similarly, high glutamine and alanine medium increased the percentage of p-STAT3–positive α cells in mouse islets transduced by the control shRNA, but not in islets-expressing *ErbB3* shRNA (Fig. 6, *D* and *E*). These results suggested that the STAT3 indeed is the downstream of amino acids activated ErbB3. Furthermore, a substantial fraction of α cells were p-STAT3 positive in islets from *Gcgr*^*−/−*^ mice but were absent in WT islets (Fig. 6, *F* and *G*), further strengthening the correlation between STAT3 activation and α cell proliferation.

To further determine whether STAT3 activation is necessary for high amino acid–induced α cell proliferation, we added STAT3 inhibitor (Stattic) to high amino acid medium during mouse islet culture. As shown in Figure 6, *H* and *I*, Stattic significantly reduced high amino acid–induced α cell proliferation. However, Stattic was less potent than the mTORC1 inhibitor rapamycin (Fig. S5, *A* and *B*). To explore the interaction between mTORC1 and STAT3 pathways, we assessed the effect of rapamycin treatment on p-STAT3 levels. Rapamycin significantly reduced p-STAT3 levels (Fig. 6, *J* and *K*). However, Stattic treatment had little effect on the percentage of pS6-positive α cells (Fig. S5, *C* and *D*), indicating that mTORC1 acts upstream of STAT3 in this setting. These results suggest STAT3 signaling activated by ErbB3 also plays a role in high amino acid–induced α cell proliferation.

### High amino acid medium activates cyclin D2 and suppresses p27 *via* ErbB3 to promote cell cycle

**Figure 7:**
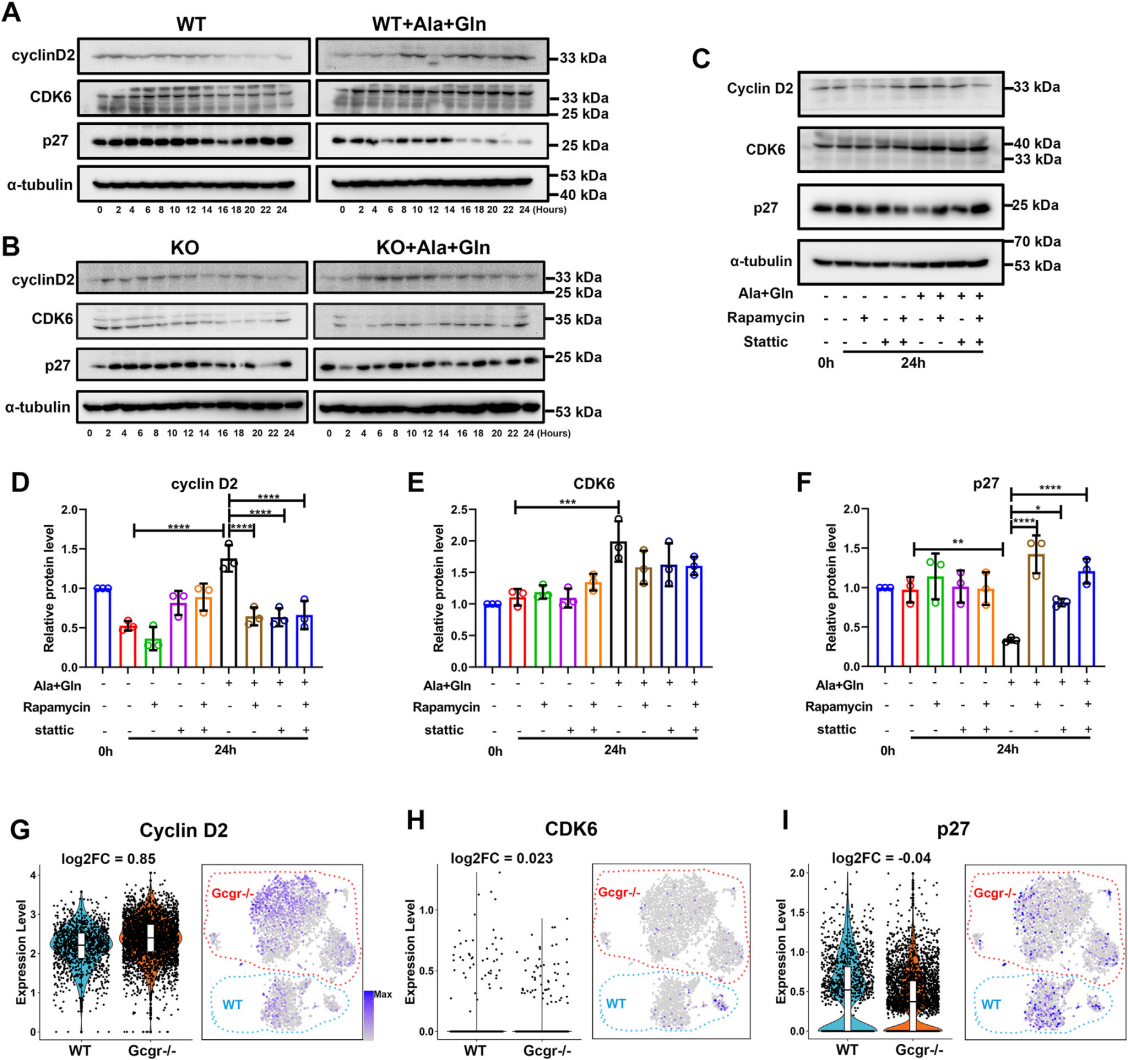
**A high amino acid medium activates cyclin D2 and suppresses p27 *via* ErbB3 to promote cell cycle.***A,* Western blot analysis of selected cell cycle regulators in WT αTC1-6 cells cultured in control medium or medium with high amino acids for 2-h increments. *B,* Western blot analysis of selected cell cycle regulators in *Erbb3* KO cultured in control medium or medium with high amino acids for 2-h increments. *C–F,* the representative images (*C*) and quantification of cyclin D2 (*D*), CDK6 (*E*), and p27 (*F*) levels in WT αTC1-6 cells cultured in the control medium or medium with high amino acids for 24 h in the presence or absence of 30 nM rapamycin and/or 1 μM Stattic. n = 3, and the relative protein level was analyzed using one-way ANOVA with a Bonferroni post hoc test. Data are presented as mean ± SD. ∗ *p* < 0.05, ∗∗*p* < 0.01, ∗∗∗*p* < 0.001, and ∗∗∗∗*p* < 0.0001. *G–I,* violin and t-distributed stochastic neighbor embedding plots showed the expression level and distribution of *cyclin D2* (*G*), *CDK6* (*H*), and *p27* (*I*) in WT and *Gcgr*^*−/−*^ mouse α cells from our single-cell RNA-seq (GSE253271). CDK, cyclin dependent kinase; GCGR, glucagon receptor.

Since activation of G1 cyclin and cyclin dependent kinases (CDKs) is critical for cell proliferation, we examined the G1 cyclins and CDKs levels upon high amino acid treatment in αTC1-6 cells. As these cells are immortalized and mitotic, we cultured them in glutamine-free minimum essential medium (MEM) with 5% serum for 24 h to inhibit cell cycle. The cells were then maintained in the same medium or supplemented with glutamine and alanine. In the following 24 h, the cells were harvested every 2 h to survey the levels of selected G1 regulators. In WT αTC1-6 cells, CDK4 was unchanged in both conditions, while CDK6 levels started to decline gradually after 16 h in the control medium but remained constant in the high amino acid medium (Figs. 7*A* and S6, *A* and *B*). Cyclin D1 was undetectable while Cyclin D3 remained unchanged during the 24 h in both control and high amino acid medium (Fig. S6*A*). In contrast, cyclin D2 gradually diminished after 14 h in the control medium but increased after 8 h of high amino acid stimulation (Figs. 7*A* and S6*B*). The CDK inhibitor p21 was unchanged in both conditions. While p27 was constant in the control medium, it declined after 16 h in the high amino acid medium (Figs. 7*A* and S6, *A* and *B*). In *ErbB3* KO αTC1-6 cells, although the high amino acid medium prevented CDK6 decline, it did not sustain cyclin D2 expression or suppress p27 as in WT cells (Figs. 7*B* and S6*B*). These data suggested that the high amino acid medium promotes cell cycle entry by upregulating CDK6 and cyclin D2 and suppressing p27, with the changes in cyclin D2 and p27 mediated by ErbB3. To assess the role of STAT3 and mTORC1 in controlling these G1 regulators, we applied rapamycin and Stattic alone or in combination to the high amino acid medium for 24 h. Both treatments significantly reduced cyclin D2 induction (Fig. 7, *C* and *D*) and blunted p27 suppression (Fig. 7, *C* and *F*). However, these treatments had little effect on CDK6 level, suggesting its independence from ErbB3 (Fig. 7, *C* and *E*). Consistent with our experimental results, our single-cell RNA-seq (GSE253271) also indicated that *cyclin D2* increased and *p27* decreased in *Gcgr*^*−/−*^ mouse α cells (Fig. 7, *G*–*I*). These results suggest that ErbB3 acts through mTOR and STAT3 to regulate cyclin D2 and p27 expression and cell cycle progression.

### The activation of ErbB3 may mediated by ErbB2/ErbB3 heterodimer

**Figure 8:**
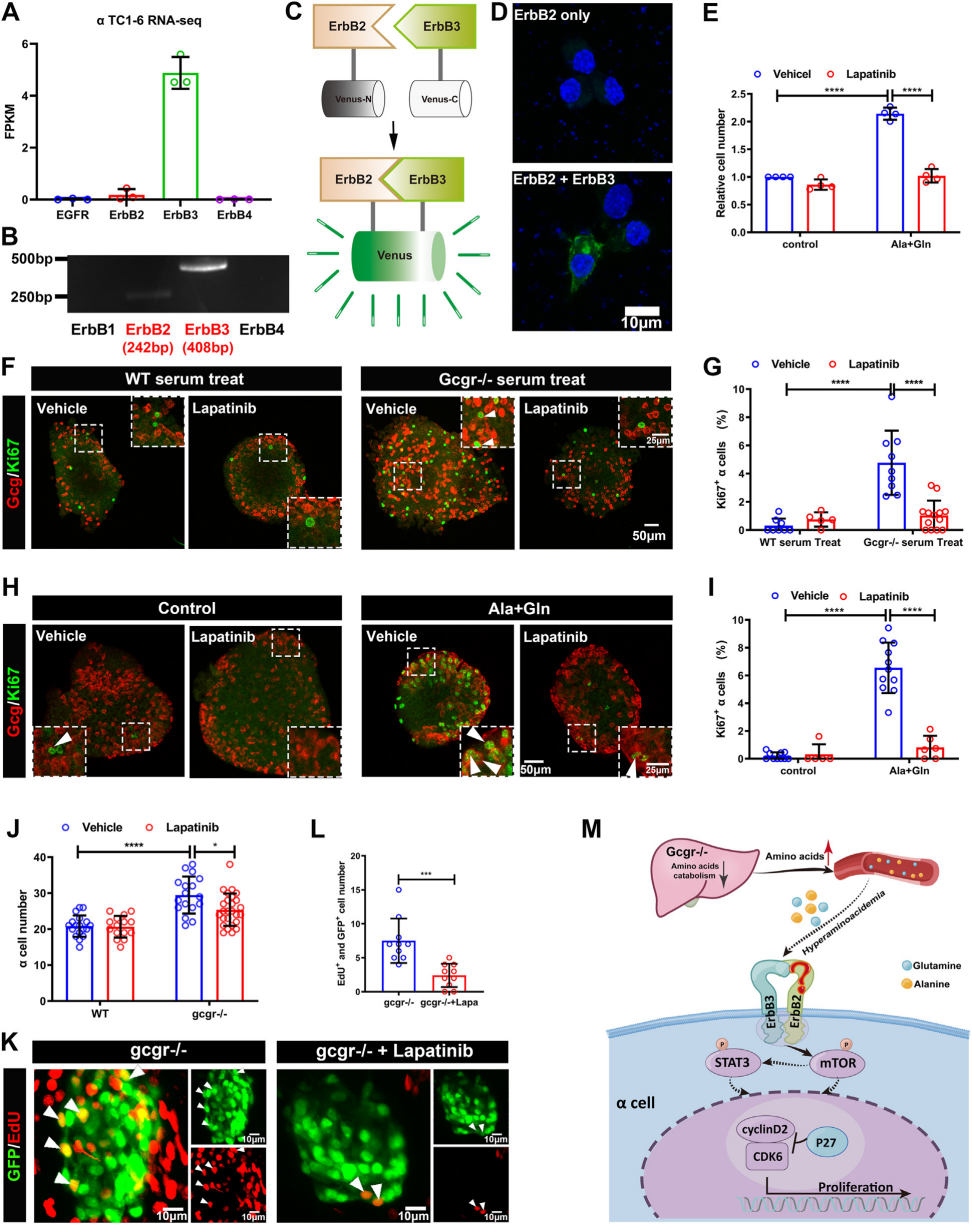
**The ErbB2 forms heterodimer with ErbB3 involved in α cell proliferation**. *A,* the *ErbBs* expression level in αTC1-6 cell line from published αTC1-6 RNA-seq data (GSE112511). *B,* the RT-PCR analysis of *ErbBs* family members expression in αTC1-6 cell. *C,* schema of bimolecular fluorescent complimentary (BiFC) assay for *ErbB2* and *ErbB3* heterodimer. The *ErbB2* carries Venus N-terminal fragment and the *ErbB3* carries Venus C-terminal fragment, the formation of *ErbB2* and *ErbB3* heterodimer leads to Venus N-terminal couple with Venus C-terminal, and shine the fluorescence of Venus finally. *D,* the representative images of Venus fluorescence caused by *ErbB2* and *ErbB3* heterodimer formation in αTC1-6, the cell only transfected with *ErbB2*-Venus-N as the negative control, and the scale bar represents 10 μm. *E,* the relative cell growth of αTC1-6 cultured in the control medium or medium supplemented with glutamine and alanine in the presence of lapatinib (5 μM) or vehicle (n = 4), and the relative cell number was analyzed using two-way ANOVA with a Bonferroni post hoc test. Data are presented as mean ± SD. ∗∗∗∗*p* < 0.0001. *F* and *G,* representative immunofluorescence images (*F*) and quantification (*G*) of islets incubated in a medium containing WT or *Gcgr*^*−/−*^ serum in the presence of lapatinib (5 μM) or vehicle (n = 5–13), the Ki67-positive α cell ratio was analyzed using two-way ANOVA with a Bonferroni post hoc test. Data are presented as mean ± SD. ∗∗∗∗*p* < 0.0001. *H* and *I,* representative immunofluorescence images (*H*) and quantification (*I*) of islets cultured in control or 4 mM glutamine and alanine contained medium in the presence of lapatinib (5 μM) or vehicle, glucagon (*red*) and Ki67 (*green*) are shown. n = 5 to 11, and the Ki67-positive α cell ratio was analyzed using two-way ANOVA with a Bonferroni post hoc test. Data are presented as mean ± SD. ∗∗∗∗*p* < 0.0001. *J,* quantification of α cell number in WT and *gcgr*^*−/−*^*; Tg(gcga: GFP)* was treated with vehicle (dimethyl sulfoxide) or lapatinib, respectively, n = 15 to 26, and the α cell number was analyzed using two-way ANOVA with a Bonferroni post hoc test. Data are presented as mean ± SD. ∗*p* < 0.05 and ∗∗∗∗*p* < 0.0001. *K* and *L,* the representative islet images (*K*) and quantification (*L*) of EdU-positive α cells in *gcgr*^*−/−*^*; Tg(gcga: GFP)* treated with or without lapatinib. α cells were shown as *green*, and EdU were shown as *red*. *Arrows* indicate double positive cells. The scale bar represents 10 μm. The α cell number was analyzed using the unpaired two-tailed *t* test (n = 10). Data are presented as mean ± SD. ∗∗∗*p* < 0.001. *M,* working model of hyperaminoacidemia-induced α cell hyperplasia through ErbB2/ErbB3 heterodimer. Elevated serum amino acid levels indirectly activate ErbB3 and its partner, triggering the downstream mechanistic target of rapamycin complex 1 and signal transducer and activator of transcription 3 signalings, which regulate cell cycle regulators to promote α cell proliferation. dpf, days post fertilization; EdU, 5-ethynyl-2-deoxyuridine; GCGR, glucagon receptor.

It is well known that ErbB3 lacks kinase activity and requires heterodimerization with other ErbBs for intracellular signal transduction. To identify the expression of other ErbBs, we analyzed the profile of ErbBs in published RNA-seq data (GSE112511) and performed RT-PCR analysis in αTC1-6 cells. As shown in Figure 8, *A* and *B*, *ErbB3* is the predominant member in αTC1-6 cells. ErbB2 is very low but still detectable, while ErbB1 and ErbB4 are undetectable. These data imply that the meager ErbB2 levels can initiate sufficient ErbB3 signaling.

To confirm the role of ErbB2 in α cell proliferation, we first tested the effect of lapatinib (EGFR and ErbB2 selective inhibitor) on glutamine- and alanine-induced αTC1-6 cell proliferation. Lapatinib significantly suppressed glutamine- and alanine-induced αTC1-6 cell proliferation (Fig. 8*E*), consistent with a functional role for ErbB2. To determine whether ErbB2 is involved in the α cell proliferation in mouse islets, we cultured islets in medium with *Gcgr*^*−/−*^ serum or high amino acids with or without lapatinib. Lapatinib-treated islets also showed a decrease of Ki67-positive α cells under *Gcgr*^*−/−*^ serum or high amino acid stimulation (Fig. 8, *F*–*I*). Finally, we tested the effect of lapatinib on the zebrafish model. As shown in Figure 8, *J*–*L*, lapatinib significantly lowered the α cell number and α cell proliferation in *gcgr*^*−/−*^ larvae. These results suggest that hyperaminoacidemia activates ErbB3 *via* ErbB2.

Taken together, these data suggest that glutamine and alanine activate ErbB2/ErbB3 heterodimers in α cells, which in turn activates mTORC1 and STAT3 to promote cell cycle progression by activating cyclin D2 and suppressing p27 (Fig. 8*M*).

## Discussion

Genetic or pharmacologic disruption of GCGR results in persistent hyperaminoacidemia, triggering α cell proliferation to overcome glucagon resistance. Despite recent advances much remains to be illuminated on the detailed mechanism of hyperaminoacidemia-induced α cell hyperplasia. In this study, we identified ErbB3 as an essential component for this response. In αTC1-6 cells, primary mouse islets, zebrafish, and mice, we show that ErbB3 is activated in α cells in conditions of high amino acid and loss of ErbB3 prevents α cell hyperplasia.

We showed a drastic increase of p-ErbB3 in the α cells in *Gcgr*^−/−^ mice and mouse islets treated with *Gcgr*^*−/−*^ serum or high amino acid (Figs. 3 and 4). Importantly, genetic suppression of *ErbB3* greatly decreased α cell proliferation in all models tested (Figs. 4 and 5). Our data suggested that ErbB3 play a critical role in hyperaminoacidemia stimulated α cell proliferation.

One surprising finding is the dependence of mTORC1 activation on ErbB3 (Figs. 5, *G*–*K* and S5). It is commonly thought that hyperaminoacidemia activates mTORC1 through increasing intracellular amino acid levels (17, 18, 19, 23). This is supported by the requirement of SLC7A2 for mTORC1 activation in α cells (27). Yet mTORC1 is activated in α cells, but not β cells, in the islets in conditions of hyperaminoacidemia (17, 18, 19). Hyperaminoacidemia activation of other signaling pathways such as the amino acid–sensitive CaSR has been reported recently to be required for mTORC1 activation in α cells (28). As CaSR is also expressed in other islet endocrine cells (51), the cell type specificity of mTORC1 activation remains unresolved. Here, we show that ErbB3 is specifically expressed in α cells, activated by hyperaminoacidemia, and required for mTORC1 activation, explaining the cell type specificity.

Downstream of ErbB3, this study also identified a role for STAT3 in high amino acid–induced α cell proliferation (Fig. 6). This adds to previous studies demonstrating two other ErbB3 downstream effectors, mTORC1 and ERK1/2, in hyperaminoacidemia-induced α cell proliferation (17, 28). STAT3 transcriptionally regulates cell cycle genes (40, 41). We showed that high glutamine and alanine increased p-STAT3 levels in αTC1-6 cells and that the nuclear p-STAT3 levels are dramatically increased in α cells in *Gcgr*^*−/−*^ mice or mouse islets treated with high glutamine and alanine (Fig. 6). Interestingly, interleukin 6–STAT3 pathway has been reported to be necessary for high-fat diet–induced α cell mass expansion (52). Our data suggest that STAT3 is directly or indirectly regulated by mTORC1. Previous studies have shown that mTORC1 activation enhances STAT3 transcriptional activity (53) and STAT3 is a direct substrate of mTORC1 (48). A similar mechanism may operate in α cells.

Hyperaminoacidemia is predicted to alter the expression of cell cycle regulators to cause α cell proliferation. We analyzed changes in the protein levels of selected G1 regulators in αTC1-6 cells at various durations of high amino acid treatment. We identified cell cycle–promoting changes in CDK6, cyclin D2, and p27 by high amino acid treatment (Fig. 7). Of these, cyclin D2 and p27 regulation is dependent on ErbB3 signaling pathway and can be canceled by inhibiting mTORC1 or STAT3. What stabilizes CDK6 downstream of high amino acid remains to be determined. Our study thus identified a role for mTOR- and STAT3-dependent upregulation of cyclin D2 and downregulation of p27 in hyperaminoacidemia-induced α cell proliferation.

One of the uncertain questions is whether other receptor tyrosine kinases may act as partners of ErbB3 to mediate the hyperaminoacidemia-induced α cell hyperplasia. Although we suggest that ErbB2/ErbB3 heterodimer mediates the signal transduction of ErbB3 activity, the level of ErbB2 in mouse α cell and αTC1-6 cells are very low. Studies have suggested that ErbB3 can form complexes with partners other than ErbB family receptors, such as insulin-like growth factor 1 receptor, fibroblast growth factor receptor 2, and c-MET (MET proto-oncogene, receptor tyrosine kinase) (54). Further studies need to elucidate the detail partners of ErbB3 in this model.

Another remaining question is how hyperaminoacidemia activates ErbB3. The expression of ErbB3 ligands *Nrg1* and *Nrg2* is unchanged or undetectable (Fig. S7), suggesting a ligand-independent mechanism. Some GPCRs can stimulate ErbB family receptors *via* transactivation in a ligand-dependent or independent fashion (55). GPCRs can activate Src family kinases (56), which directly phosphorylate ErbB family and other receptor tyrosine kinases (57, 58). We previously reported that activation of a Gq-DREADD leads to mTORC1 and ERK1/2 activation in α cells (28). ErbB3 may be a downstream effector of Gq signaling in α cells through transactivation.

In conclusion, our study demonstrated ErbB3 as a crucial mediator of hyperaminoacidemia-induced α cell proliferation. Activation of ErbB3 stimulates mTOR and STAT3, which contribute to the upregulation of cyclin D2 and downregulation of p27, leading to α cell proliferation. Our study provided a new component in the pathway from hyperaminoacidemia to α cell proliferation. The results may also help better control α cell proliferation for diabetes therapy.

## Experimental procedures

### Mouse models

All animal experiments were approved by the Xiamen University Institutional Animal Care and Use Committee. C57BL/6 WT mice were purchased from Xiamen University Laboratory Animal Center. *Gcgr*^*−/−*^ mouse was originally generated from Xiamen University Laboratory Animal Center (14). Male 10 to 12 weeks old mice were used for all mice experiments, all mice were bred and maintained in a standard pathogen-free environment under standard rodent chow and 12 h light/12 h dark cycle of the Laboratory Animal Center in Xiamen University, and all animal experiments were approved by the Animal Care and Use Committee of Xiamen University (Protocol XMULAC20160089, 10 March 2016).

### Zebrafish models

Zebrafish (*Danio rerio*) were raised in a recirculating aquaculture system (Shanghai Haisheng Biotech Co., Ltd) with a consistent 14:10 h light-dark cycle. Embryos were obtained by natural breeding and maintained in embryo rearing solution at 28.5 °C and staged according to a standard protocol (59). In this study, the *gcgra*^*−/−*^*, gcgrb*^*−/−*^ double mutant line (referred as *gcgr*^*−/−*^) (32), and *Tg(gcga:GFP)* (60) were used. All procedures had been approved by the Institutional Animal Care and Use Committee at Xiamen University (Protocol XMULAC20160089, 10 March 2016) and Vanderbilt University (M/14/215).

The zebrafish *gcgr*^*−/−*^*; Tg(gcga: GFP)* for drug screening were from previously study (32). The *Tg(gcga: CD533;LC)* line was generated by the Tol2 transposon system (61). The CD533 fragment was inserted by the ClaI and Xbal site into pME-MCS. The transgenic construct was assembled using a multisite Gateway LR reaction consisting of p5E-gcga, pME-CD533, p3E-polyA, and pDestTol2-LC (62). pDestTol2-LC carries an α crystallin: mCerulean reporter, allowing identification of transgenic fish as they express CFP in the lens.

The *erbB2* and *erbB3b* knockdown in zebrafish larvae was achieved by CRISPR-Cas9. The guide sequences were selected from CRISPRscan (63). The following targets were selected (PAM underlined): ErbB2, GAGATAGTGGAGGTTCAGGGG, and CTGAGAGAACTTCGCCTCAGG; ErbB3b, CCTCATTGCGATGAACCAGTT, and CCCTAATGAATGCTGCCACCC. The sgRNAs were synthesized by a T7 *in vitro* transcription kit (Invitrogen, AM1314). Two sgRNAs (50 ng/μl) targeting each gene were incubated with Cas9 protein (2 μM, NEB, M0646) and microinjected into the one-cell stage zebrafish eggs. The knockdown efficiency was tested by a heteroduplex mobility assay as described previously (64).

### Cell line

α TC1 clone 6 (American Type Culture Collection CRL-2934) was from American Type Culture Collection and cultured in Dulbecco's modified Eagle's medium (Gibco, C11885500) containing 10% fetal bovine serum (FBS) and 100 U/ml penicillin, 100 μg/ml streptomycin, 10 mM Hepes, and incubated in a cell culture incubator at 37 °C with 5% CO_2_.

### Small molecule compound screening

The *gcgr*^*−/−*^*;Tg(gcga: GFP)* zebrafish that display α cell hyperplasia by 4 dpf were used for compounds screens (32). The embryos were maintained in 0.3X Daniel solution. At the 4 dpf, the larvae were transferred into 24-well plates and incubated with 10 μM individual compounds for 3 days. Compounds from the GSK kinase inhibitor library (349 compounds, https://www.microsoft.com/en-us/microsoft-365/excel) and the Enzo kinase inhibitor library (80 compounds) were provided by the Vanderbilt high-throughput screening core. At the 7 dpf, the larvae were harvested and their α cell numbers were counted under a fluorescence microscope, and then the Z-score value was calculated in Microsoft Excel.

### Zebrafish islet and α cells isolation; RT-PCR

To isolate islets, 7 dpf *Tg(gcga: GFP)* larvae were euthanized, mildly digested with collagenase P (1.2 mg/ml, Sigma-Aldrich) for 5 min at 37 °C in a water bath, and gently homogenized by pipetting up and down. The islets were picked manually under a fluorescence microscope (Lecia M205 FCA, Lecia Wetzlar) from the homogenate. The islets were digested to single cell suspension by Liberase DH (100 μg/ml, Roche) for 50 min, at 36 °C. The α cells were gathered by using fluorescence-activated cell sorting (BD AriaIII) into an Eppendorf tube containing TRIzol reagent (Thermo Fisher Scientific) for RNA extraction according to the manufacturer’s instruction. The complementary DNA was synthesized using M-MLV reverse transcriptase (Promega). RT-PCR was performed to analyze *erbB* family expression levels in sorted α cells. For RT-PCR analysis of mouse *ErbB* family members, RNA was extracted from αTC1-6 cells. Primers are listed in Table S2.

### EdU labeling and detection in zebrafish larvae

To identify proliferating α cells, zebrafish larvae were treated with 1 mM 5-ethynyl-2-deoxyuridine (EdU) at 6 dpf for 24 h. The EdU signal was detected by Click-It EdU Alexa Fluor 594 imaging kit (C10339; Invitrogen) according to the manufacturer’s protocol. Islet images were captured using a Leica SP8 microscope.

### Immunofluorescence in mouse cryosections and islet

For the mouse pancreas cryosections, 10- to 12-week-old male mice were euthanized and the pancreata harvested. The pancreata were fixed in 4% paraformaldehyde (PFA) and submerged in 30% sucrose solution. The pancreata were embedded in optimal cutting temperature compound. Ten micrometers cryosections were stained with a primary antibody (Table. S3) and a corresponding second antibody (Table. S3) as previously described (65, 66).

For whole islet immunofluorescence, islets were fixed with 4% PFA in a cell culture plate. After permeabilization with 0.3% Triton X-100 for 3 h, the islets were incubated with a primary antibody and the appropriate secondary antibody. The islets were transferred to a glass slide for imaging. Islets images were captured using a Leica SP8 confocal microscope.

### Mouse islet isolation and serum/amino acids treatment

Mouse islets were isolated from 10- to 12-week-old male C57BL/6J mice. After euthanasia, pancreas was perfused with collagenase type IV (1 mg/ml, Thermo Fisher Scientific, 17104019) and harvested to digestion at 37 °C for 15 min. Islets were purified in a histopaque density gradient (Sigma, 10771). All islets were picked manually under a microscope. Islets were cultured in RPMI 1640 (Gibco, C11875500) containing 10% FBS and 1% penicillin/streptomycin. A final concentration of 10% of WT or *Gcgr*^*−/−*^ serum was used to test their effects on α cell proliferation. To determine the role of amino acids, glutamine- and alanine-free RPMI 1640 was used and glutamine and/or alanine was added to the desired final concentration. To assay the effect of inhibitors, rapamycin (MCE, 10219) (30 nM) and Stattic (MCE, 13818) (1 μM) were added simultaneously to the final concentration with amino acids. All islets were cultured for 6 days.

### ErbB3 shRNA adenovirus generation and transfection

The recombinant adenoviruses were generated using the AdEasy system (67). In brief, the sh-target was inserted into pShuttle-U6 with CMV-GFP at the XbaI and SaII sites. The *ErbB3* targeting sequence is as follows: CGTGTCTACATAAGTGCCAAT. The target containing plasmid was linearized and transformed into AdEasier competent cells. After the recombination reaction, the positive clones were selected for plasmid extraction, and the final recombinant plasmid was linearized and transfected into 293A cells to produce adenoviruses. The empty vector was used as the control.

For the adenovirus transfection, the sh-ErbB3 and control adenoviruses were present throughout. GFP signal in islets was the indicator for transfection efficiency. All islets were cultured for 6 days.

### Generation of ErbB3 KO αTC1-6 subclone

The *ErbB3* was mutated in αTC1-6 by CRISPR-Cas9. The sgRNA target sequence (TGCTCTGCGCCAGCTCCGGT) was selected from CHOPCHOP (68) website. The target was assembled into PX459 vector by BbsI-based golden gate ligation (NEB, New England Biolabs). The resultant plasmid was transfected into αTC1-6 by Lipofectamine 2000 (11668500, Thermo Fisher Scientific). The transfected cells were screened under 2.5 μg/ml puromycin (Sangon Biotech). Positive clones were verified by sequencing of the targeted region and Western blot.

### αTC1-6 cell proliferation assay

To assay amino acid stimulated αTC1-6 cell (both WT and ErbB3 KO) proliferation using the CCK8 kit (GLPBIO, GK10001), cells were seeded in 96-well plate in MEM (Gibco, 11090-081) supplemented with 5% FBS, 1% penicillin/streptomycin with glutamine (4 mM), and/or alanine (4 mM). The assay was performed every 24 h from day 1 to 5. The absorbance at 450 nm was measured by a plate reader (GloMax Discover, Promega).

### Bimolecular fluorescent complimentary assay

The ErbB2 and ErbB3 CDS region were cloned into the pcDNA 3.1-Venus-N and pcDNA 3.1-Venus-C, respectively, after KpnI and EcoRI digestion (NEB, New England Biolabs) using a One Step Cloning Kit (Yeasen, 10911ES20). The resultant two plasmids were cotransfected into αTC1-6 cells by Lipofectamine 2000 (11668500, Thermo Fisher Scientific). Cells transfected with ErbB2-Venus-N-Erbb2 alone served as a negative control. After 24 h, the transfected cells were fixed with 4% PFA and stained with 4′,6-diamidino-2-phenylindole (Beyotime, C1002). Cell images were captured with a Leica SP8 confocal microscope.

### Western blotting

For the amino acids treatment experiment, the αTC1-6 cells were incubated with alanine (4 mM) and glutamine (4 mM) replenished for 48 h in MEM (Gibco, 11090-081) supplemented with 5% FBS and 1% penicillin/streptomycin. To determine the levels of selected cell cycle regulators, αTC1-6 cells were cultured in glutamine-free MEM with 5% FBS and 1% penicillin/streptomycin for 24 h to inhibit proliferation. Glutamine and/or alanine were then added. In some experiments, rapamycin (30 nM) and Stattic (1 μM) were added together with amino acids. The cells were harvested every 2 h until 24 h of treatment. The cells were lysed immediately in a commercial radioimmunoprecipitation assay lysis buffer (Beyotime P0013C: 50 mM Tris (pH 7.4), 150 mM NaCl, 1% NP-40, 0.5% sodium deoxycholate), the protease inhibitor cocktail (MCE, HY-K0010) and phosphatase inhibitor cocktail (MCE, HY-K0021) were added in radioimmunoprecipitation assay buffer before using. After determining the protein concentration, the lysates were boiled in SDS containing loading buffer. An equal amount of protein was loaded per lane in an 8 to 10% SDS-polyacrylamide gel. The samples were resolved by electrophoresis and transferred to a polyvinylidene fluoride membrane. After blocking with 5% fat-free milk, the membrane was incubated with a primary antibody (Table. S3), followed by an appropriate horseradish peroxidase–conjugated secondary antibody (Table. S3). Finally, the horseradish peroxidase–conjugated secondary antibody on the membrane was detected by an enhanced chemiluminescence solution (Advansta, K-12045-D50). The solution was added to the preprepared polyvinylidene fluoride membrane. The chemiluminescence signal was detected by an enhanced chemiluminescence system (Bio-Rad). Images were acquired after appropriate exposure. The captured images were analyzed by Image Lab software (Bio-Rad, https://www.bio-rad.com/en-cn/product/image-lab-software?ID=KRE6P5E8Z). The signal intensity of the phospho-protein level was normalized to that of corresponding total protein, and the total protein was normalized to the loading control protein (GAPDH or α-tubulin). The signal intensity of cell cycle regulators was normalized to that of α-tubulin.

### Statistical analysis

For the zebrafish α cell counting, n indicates the number of larvae, for the quantitative reverse transcription polymerase chain reaction and Western blotting, and n indicates the biological sample repetition. For the α cell ratio statistical analysis, n indicates the islet number, and α cells in individual islets were included in statistics. For the α cell ratio in pancreas sections, n represents the number of sections. Details were shown in Figure legends.

All statistical analyses were performed using GraphPad Prism v.9.0 (GraphPad Software, https://www.graphpad.com/updates/prism-900-release-notes). Results are presented as mean values ± SD. In multigroup comparison with one independent variable, one-way ANOVA was used followed by Bonferroni post hoc test or *t* test. In multigroup comparison with two independent variables, two-way ANOVA was performed followed by Bonferroni post hoc test or *t* test. For the comparison of two independent groups, the unpaired two-tailed *t* test was used. *p* < 0.05 was considered as significant (∗*p* < 0.05, ∗∗*p* < 0.01, ∗∗∗*p* < 0.001, and ∗∗∗∗*p* < 0.0001).

## Data availability

All data supporting the findings of this study are available within the article. Any further information is available from the corresponding author upon reasonable request.

## Supporting information

This article contains supporting information.

## Conflict of interest

The authors declare that they have no conflicts of interest with the contents of this article.
