# Supplementary material for: ErbB3 is required for hyperaminoacidemia-induced pancreatic α cell hyperplasia
Source: J Biol Chem. 2024 Jun 27;300(8):107499. doi: 10.1016/j.jbc.2024.107499 (PMC11326907; doi:10.1016/j.jbc.2024.107499)
Supplement: Supplementary Materials [file mmc1.docx]

**Supplementary data**

**Supplementary Research design and methods (method only used in supplemental data)**

**αTC1-6 cell EdU labeling**

To assay proliferation by EdU labeling, αTC1-6 cells were cultured in a medium supplemented with 10% WT or *Gcgr^-/-^* mouse serum for 24 hours. EdU (Beyotime, C0071S and C0078S) was added to 10 µM 2 hours before the cells were fixed. EdU was detected according to the manufacturer’s instructions. The images were captured by a Leica SP8 confocal microscope and analyzed using ImageJ software (National Institutes of Health).

**NRG2 Elisa for mouse serum**

For the mouse serum NRG2 level measurement, mouse sera were collected as previously described (19), and the serum NRG2 levels were determined using a Elisa kit (Jianglai biology, JL52419) according to the manufacturer’s protocol.

**Supplementary table**

**Table S1. Results of small molecule screening in zebrafish using GSK and Enzo kinase inhibitor library.**

**Table S2: Primers for RT-PCR and qRT-PCR.**

**Table S3: Antibody used in this study**

**Table S2: Primers for RT-PCR and qRT-PCR**

| **Species** | **Gene** | **Name** | **Nucleotide sequence (5’—3’)** | **Use** |
| --- | --- | --- | --- | --- |
| Zebrafish | *ErbB1a* | F | ACGCCTCTTCAAGTCCAAACAAC | RT-PCR |
|  |  | R | GCCAGTAGACCTCCGACAACG | RT-PCR |
|  | *ErbB1b* | F | GCCCCCATAGCTTTGTAGTGACT | RT-PCR |
|  |  | R | GCACTGAGGGATTTGAGGTGTTC | RT-PCR |
|  | *ErbB2* | F | CGCTGGGATTGCGTTCTTTA | RT-PCR |
|  |  | R | CCGCACTGACCCGTGTAGGA | RT-PCR |
|  | *ErbB3a* | F | TCATTGAGTTTCCTCCTTCCACC | RT-PCR |
|  |  | R | CATCTGCACTACAAATCCCATC | RT-PCR |
|  | *ErbB3b* | F | AGAAGTCATTCCCGTGTCCG | RT-PCR |
|  |  | R | GTACTCTTGCGTGGCATCTGTT | RT-PCR |
|  | *ErbB4a* | F | ATCCGAGGCACTAAACTCTACG | RT-PCR |
|  |  | R | GTCTTTGGGTCCGAAACAGC | RT-PCR |
|  | *ErbB4b* | F | CTGATCCGCAACAACCGTGAC | RT-PCR |
|  |  | R | CTGCTCGCTCCCTGTAGACC | RT-PCR |
| Mouse | *EGFR* | F | CTGTCGCAAAGTTTGTAATGG | RT-PCR |
|  |  | R | GCAGCCTTCCGAGGAGCATA | RT-PCR |
|  | *ErbB2* | F | CAAAGAAATCCTAGATGAAGCG | RT-PCR |
|  |  | R | CCCTGTGAACAAGCCGAACT | RT-PCR |
| Mouse | *ErbB3* | F | AGGTGCCAAAGGTCCAATCTA | RT-PCR |
|  |  | R | CCCTCGGGAATCCAAATCC | RT-PCR |
|  | *ErbB4* | F | TCCACTTTACCACAACACGCTAG | RT-PCR |
|  |  | R | GCCAAGGACCTTTACCCTCT | RT-PCR |
|  | *Nrg1* | F | ATCTGTATCGCCCTGTTGGT | QRT-PCR |
|  |  | R | TCTGGTGGTGGGTTTGGAT | QRT-PCR |
|  | *Nrg2* | F | CAAGCTGAAGAAGATGAAGAGCCAGAC | QRT-PCR |
|  |  | R | CACCCTCACTTTGTTGAACTGTAGCC | QRT-PCR |

**Table S3: Antibody used in this study**

| **Antibodies** | **Source** | **Identifier** |
| --- | --- | --- |
| Rabbit anti-Egfr | Proteintech | 18986 |
| Rabbit anti-ErbB2 | Proteintech | 18299 |
| Mouse anti-ErbB3 | Santa Cruz | sc-7390 |
| Mouse anti-ErbB4 | HUABIO | RT-1276 |
| Rabbit anti-p-ErbB3 (Tyr1289) | Abcam | ab101407 |
| Guinea pig anti-insulin | Dako | A0954 |
| Mouse anti-glucagon | Sigma | G2654 |
| Rabbit anti-glucagon | Abcam | Ab92517 |
| Rabbit anti-ki67 | Abcam | Ab15580 |
| Rabbit anti-p-mTOR (Ser2448) | Affinity | AF3308 |
| Mouse anti-mTOR | Santa Cruz | sc-517464 |
| Rabbit anti-p-STAT3 (ser727) | Affinity | AF3294 |
| Rabbit anti-STAT3 | Affinity | AF6294 |
| Rabbit anti-CDK6 | Abclonal | A1545 |
| Rabbit anti-CDK4 | Abclonal | A0366 |
| Rabbit anti-cyclin D1 | Abclonal | A19038 |
| Rabbit anti-cyclin D2 | Abclonal | A13284 |
| Rabbit anti-cyclin D3 | Abclonal | A3989 |
| Rabbit anti-p27 | Abclonal | A19095 |
| Rabbit anti-p21 | Abclonal | A1483 |
| Mouse-anti-α-tubulin | Abcam | Ab7291 |
| Mouse-anti-GAPDH | Proteintech | 60004 |
| Rabbit anti-Phospho-S6(Ser235/236) | Cell Signal Technology | 4858T |
| Goat anti-Mouse IgG (H+L) Cross-Adsorbed Secondary Antibody, Alexa Fluor™ 488 | ThermoFisher | A11001 |
| Goat anti-Rabbit IgG (H+L) Cross-Adsorbed Secondary Antibody, Alexa Fluor™ 488 | ThermoFisher | A11008 |
| Goat anti-Guinea Pig IgG (H+L) Highly Cross-Adsorbed Secondary Antibody, Alexa Fluor™ 568 | ThermoFisher | A11075 |
| Goat anti-Rabbit IgG (H+L) Cross-Adsorbed Secondary Antibody, Alexa Fluor™ 647 | ThermoFisher | A21244 |
| Goat anti-Mouse IgG (H+L) Cross-Adsorbed Secondary Antibody, Alexa Fluor™ 647 | ThermoFisher | A21235 |
| Goat anti-Mouse IgG (H+L) Cross-Adsorbed Secondary Antibody, Alexa Fluor™ 568 | ThermoFisher | A11004 |
| Peroxidase-Conjugated Goat Anti-Rabbit IgG(H+L) | Yeasen | 33101ES60 |
| Peroxidase-Conjugated Goat Anti-Mouse IgG(H+L) | Yeasen | 33201ES60 |

**Supplementary Figures and figure legends.**

**Figure S1**


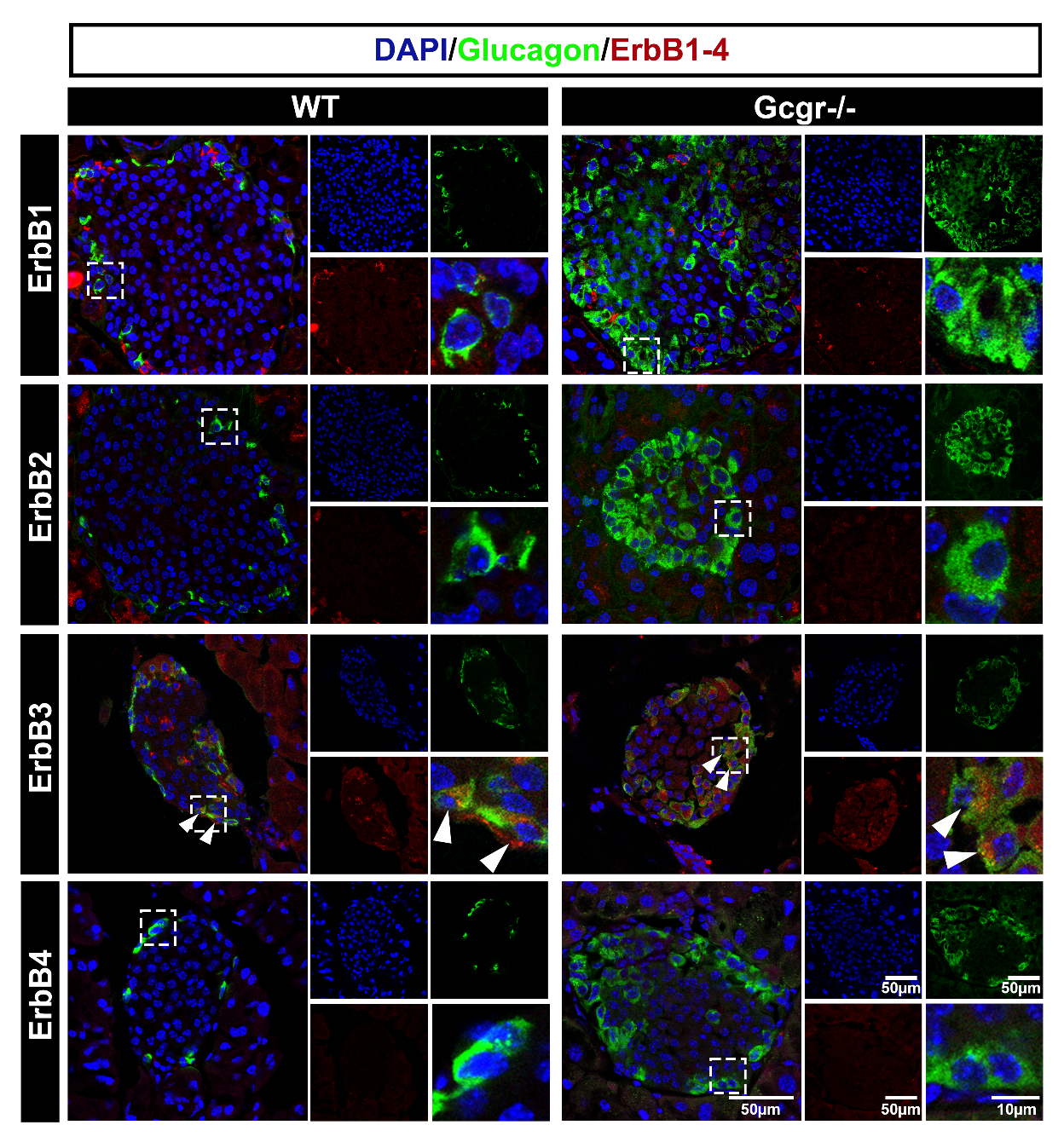


**Figure S1.** **The levels of ErbB1-4 in α cells from WT and *Gcgr^-/-^* mouse.**

Representative immunofluorescence images of ErbB1-4 in pancreatic islets from WT and *Gcgr^-/-^* mouse. DAPI (blue), insulin (red), glucagon (green), ErbB1-4 (red) are shown. Scale bar represents 50μm or 10μm as shown in the figures.

**Figure S2**


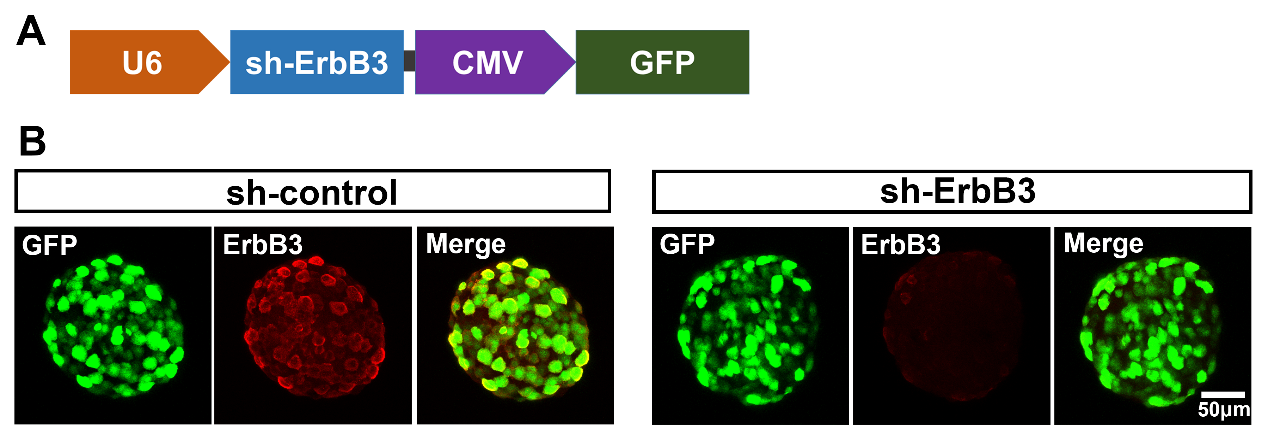


**Figure S2. Conformation of *ErbB3* knockdown by adenoviral expression of sh-RNA in mouse islets.**

(A) Schematic diagram of *ErbB3* sh-RNA-expressing adenoviral vector. (B) Representative immunofluorescence images of islets transfected with sh-ErbB3 or control adenovirus. GFP marks transduced cells. The intensity of ErbB3 immunofluorescence signal indicates its expression levels.

**Figure S3**


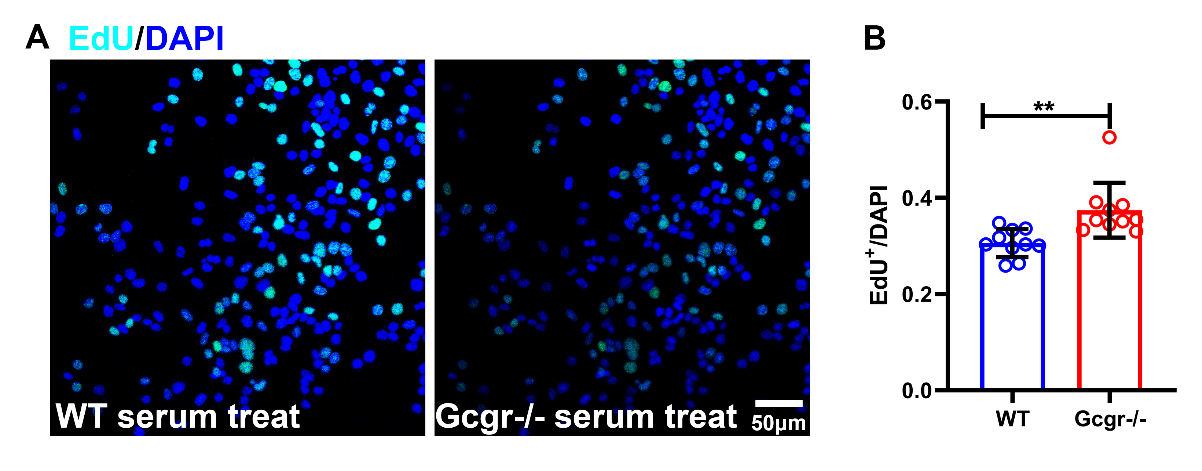


**Figure S3. *Gcgr^-/-^* mouse serum stimulates α-cell proliferation in αTC1-6 cells.**

(A and B) The representative images (A) and quantification (B) of EdU positive cells in αTC1-6 cells incubated with *Gcgr^-/-^* or WT serum for 24 hours. n = 10. The ratios of proliferating α cells were compared using unpaired two-tailed t-test. Data are presented as mean ± SD. ***p*<0.01.

**Figure S4**


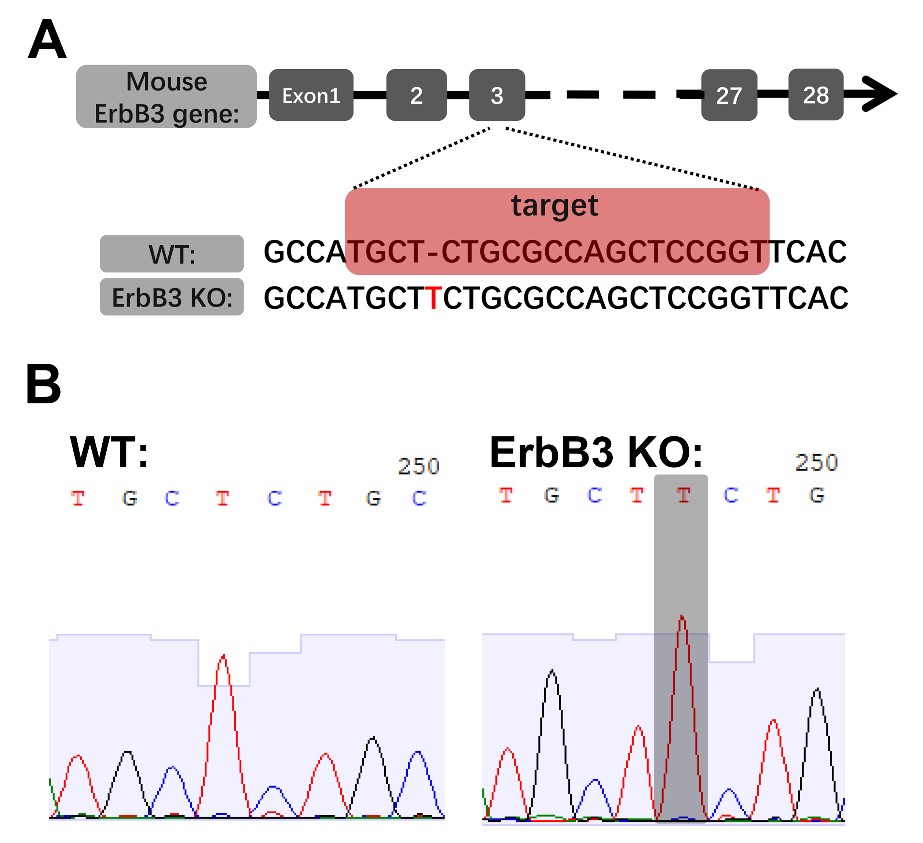


**Figure S4. Conformation of *ErbB3* KO in the mutant αTC1-6 cells.**

(A) Schematic diagram of *ErbB3* targeting, the red rectangle indicates the Crispr target, and the red letter represents the mutated base. (B) The Sanger sequencing traces of the PCR products from WT and *ErbB3* KO cells. The T insertion is highlighted grey.

**Figure S5**

**
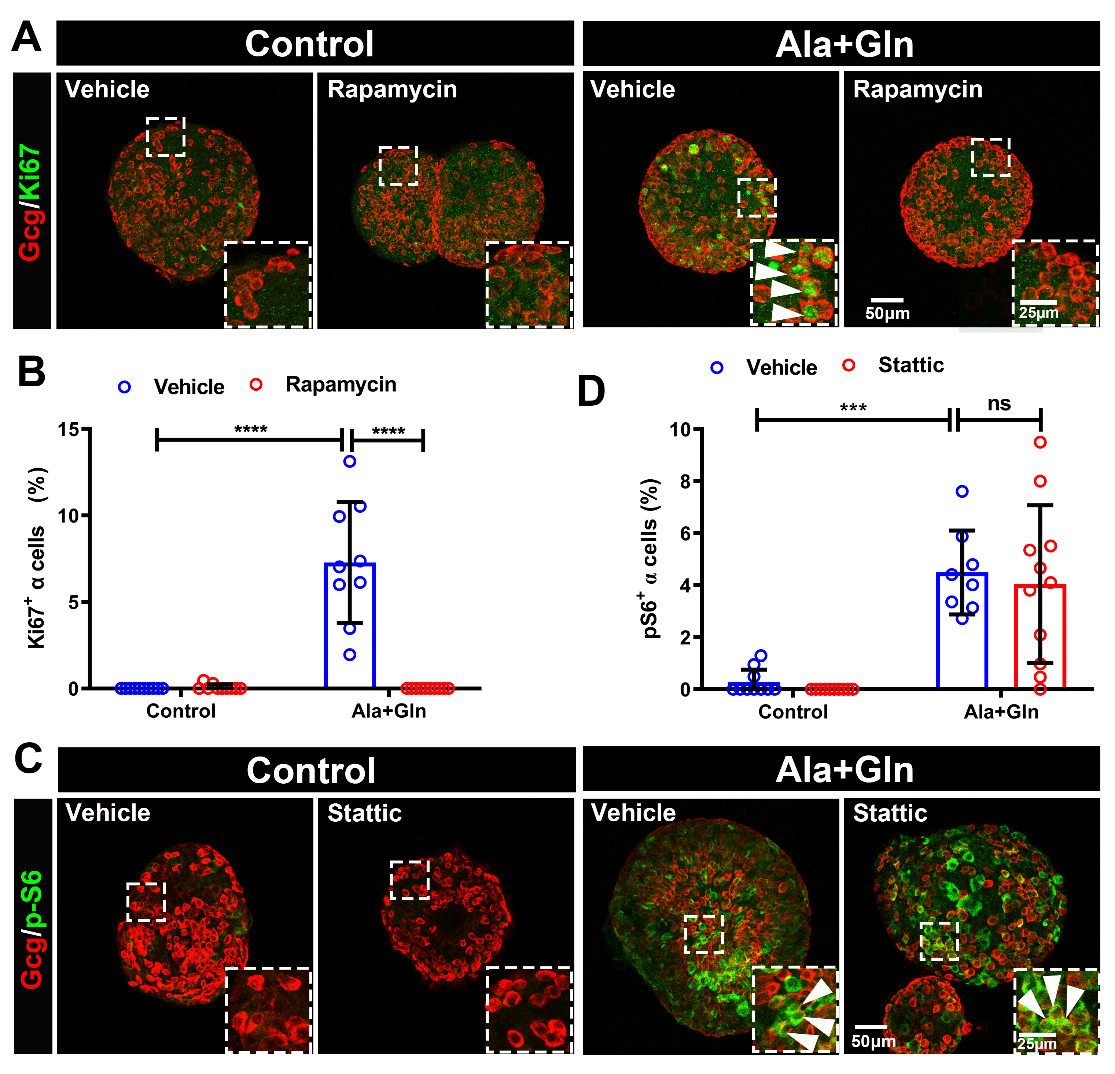
**

**Figure S5. Hyperaminoacidemia increases mTORC1 activities in α cells *in vivo* and *ex vivo* independent of STAT3.**

(A and B) Representative immunofluorescence images (A) and quantification (B) of islets cultured in the control or the 4 mM glutamine and alanine-containing medium with or without rapamycin (30nM). Glucagon (red) and Ki67 (green) are shown, n=9-10. The Ki67 positive α cell ratios were compared using two-way ANOVA with a Bonferroni post-hoc test. Data are presented as mean ± SD. *****p*<0.0001. (C and D) Representative immunofluorescence images (C) and quantification (D) of islets cultured in the control or the 4 mM glutamine and alanine-containing medium with or without Stattic (1μM). Glucagon (red) and pS6 (green) are shown. n=8-11. The p-S6 positive α cell ratios were compared using two-way ANOVA with a Bonferroni post-hoc test. Data are presented as mean ± SD. ****p*<0.001.

**Figure S6**


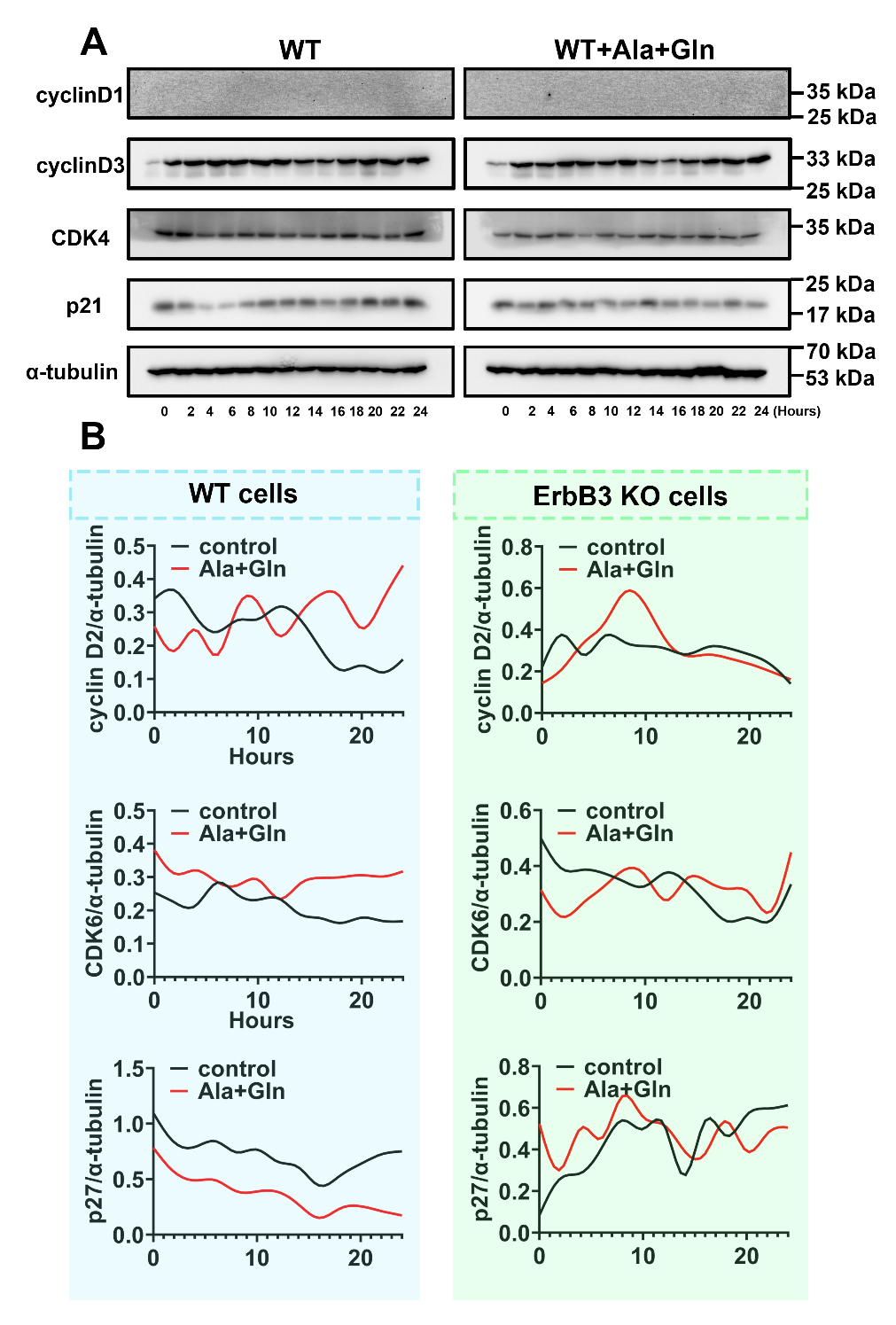


**Figure S6. Glutamine and alanine change the levels of cyclin D2, CDK6, and p27, but not cyclin D1/D3, CDK4, or p21.**

(A)The protein levels of cyclin D1, cyclin D3, CDK4, and p21 in wild-type αTC1-6 cells during a 24-hour incubation with the control or the high amino acids medium after serum limitation treatment. (B)The quantification of cyclin D2, CDK6, and p27 protein levels in wild-type and *ErbB3* knockout αTC1-6 cells in Figures 7A and 7B.

**Figure S7**


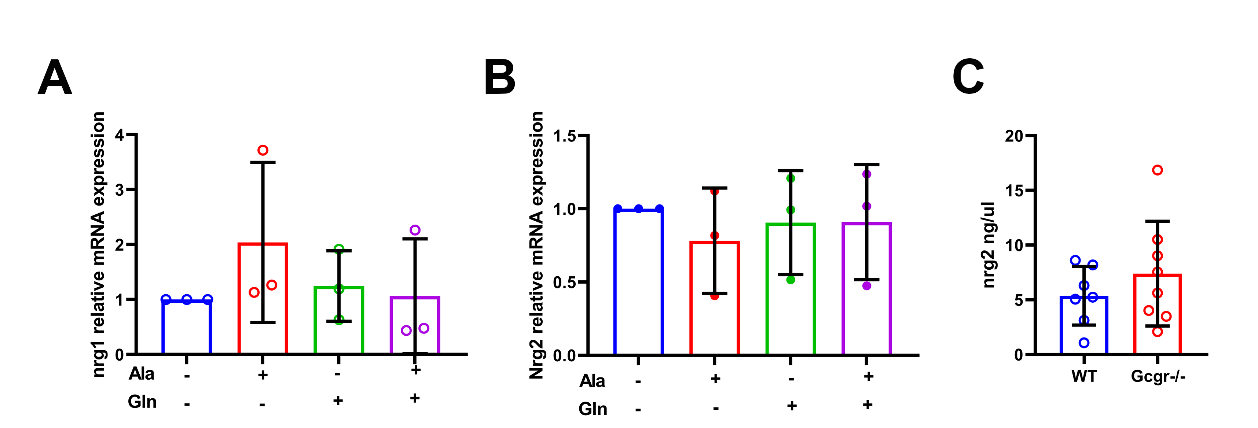


**Figure S7. High amino acid treatment does not alter the levels of *Nrg1* and *Nrg2* mRNA or serum NRG2.**

(A to B), The *Nrg1* (A) and *Nrg2* (B) mRNA levels in αTC1-6 cells after alanine and (or) glutamine treatment (n=3). The relative mRNA expression levels were compared using one-way ANOVA with a Bonferroni post-hoc test, Data are presented as mean ± SD. C, The WT and *Gcgr^-/-^* mouse serum Nrg2 level measured by Elisa kit (n=7-8). The NRG2 levels were analyzed using unpaired two-tailed t-test. Data are presented as mean±SD. The NRG1 was undetectable in mouse serum by Elisa kit.
